# Cervicovaginal microbiome composition and absolute quantity are associated with pelvic inflammatory disease

**DOI:** 10.1099/mgen.0.001574

**Published:** 2025-12-05

**Authors:** Laurence Don Wai Luu, Ciara Bryant, James Brown, Mark Turner, Thi Huong Pham, Rami Mazraani, Catherine Burke, Brittany Jury, Manisha Shrestha, Kirsty Fleming, Deborah Bateson, Darren Russell, Faith Bassett, Evonne Ong, Jane S. Hocking, Sally Sweeney, Wilhelmina May Huston

**Affiliations:** 1 School of Life Sciences, Faculty of Science, University of Technology Sydney, Ultimo, NSW, Australia; 2 School of Biotechnology and Biomolecular Sciences, Faculty of Science, University of New South Wales, Kensington, NSW, Australia; 3 School of Mathematical and Physical Sciences, Faculty of Science, University of Technology Sydney, Ultimo, NSW, Australia; 4 School of Agriculture and Food Sustainability, Faculty of Science, The University of Queensland, St Lucia, QLD, Australia; 5 Family Planning Australia, Newington, NSW, Australia; 6 The Daffodil Centre, A Joint Venture between Cancer Council NSW and the University of Sydney, Sydney, Australia; 7 Cairns Sexual Health Service, QLD Health, Cairns North, QLD, Australia; 8 Melbourne School of Population and Global Health, The University of Melbourne, Parkville, VIC, Australia; 9 Faculty of Science, University of Technology Sydney, Sydney, NSW, Australia; 10 School of BioSciences, Faculty of Science, University of Melbourne, Melbourne, VIC, Australia

**Keywords:** antibiotic, cervical microbiome, dysbiosis, *Lactobacillus*, reproductive infection, sexually transmitted infection, vaginal microbiome

## Abstract

Pelvic inflammatory disease (PID), which involves infection and inflammation of the female reproductive tract, can lead to sequelae including chronic pelvic pain, ectopic pregnancy and tubal factor infertility. A causative pathogen is not identified in many PID cases (idiopathic PID) and does not develop in all women with a sexually transmitted infection or bacterial vaginosis. Therefore, there is a need to better understand the pathogenesis of PID. A case–control study was conducted to explore microbiome, antibiotic resistance and immune gene expression in PID. Microbial profiling using both 16S rRNA gene amplicon and metagenomic approaches revealed that bacterial vaginosis-associated bacteria such as *Gardnerella vaginalis*, *Fannyhessea vaginae*, *Ureaplasma parvum* and members of the *Prevotella* spp. were significantly enriched in PID cases, while healthy controls were associated with *Lactobacillus* (*L*.) *crispatus*. Quantitative analysis with species-specific quantitative real-time PCR (qPCR) indicated that a high copy number of *L. crispatus* (measured using calibrated copy estimates by qPCR) was strongly associated with cervical samples from women in the control group, whereas PID cases with this organism had low copies when measured using qPCR. Antibiotic resistance to tetracyclines was more frequently predicted in metagenome-assembled genomes from PID cases, and corresponding isolates cultured from cases were less susceptible to doxycycline (*L. iners*). Overall, this study supports that PID is associated with cervicovaginal dysbiosis and an absence or low quantity of *L. crispatus*.

**Impact Statement**

A dysbiotic cervicovaginal microbiome has been associated with PID. Here, we examined the microbiome in PID using a case–control study design. A low copy number abundance of *Lactobacillus crispatus* was associated with PID cases. Metagenome-assembled genomes, whole-genome sequencing and cultured isolates susceptibility analysis supported that reduced antibiotic susceptibility, especially to tetracyclines, was more frequently identified in PID cases.

## Data Summary

All data generated or analysed during this study are available from ENA under the project accession numbers PRJEB80350, PRJEB80512 and PRJEB80538 or are included in this published article and its supplementary information files. A summary of all materials provided in the project is listed in Table S2.

## Introduction

Pelvic inflammatory disease (PID) is a serious gynaecological disease involving infection and inflammation of the female upper reproductive tract [1] and can result in chronic pelvic pain, ectopic pregnancy and tubal factor infertility [2,4]. PID symptoms can include acute pelvic pain, abnormal vaginal discharge or bleeding, dyspareunia and clinical findings of cervical motion, uterine and adnexal tenderness on bimanual examination [1,5]. The prevalence of PID globally was estimated to be 53.19 per 100,000 (age-standardized) [6], but this is likely an underestimate, as current PID diagnosis is based on clinical features and misses up to 80% of sub-clinical PID cases [7]. There is no molecular diagnostic test for PID, and the gold standard is considered to be laparoscopy, which is not commonly available in outpatient settings where most PID cases are diagnosed and treated [8]. Treatment for PID involves empirical combinations of broad-spectrum antibiotics [including ceftriaxone, doxycycline (DOX) and metronidazole] [9], with a low threshold of suspicion required to treat, given the risks associated with serious sequelae.

PID can be caused by sexually transmitted infections (STIs), uterine instrumentation and gynaecological procedures. In many cases, there is no known cause identified, and this is referred to as idiopathic PID (50–60% of cases) and often involves bacterial vaginosis (BV)-associated organisms [10,11]. In STI-associated PID, infection of the lower reproductive tract with STIs including *Chlamydia* (*C*.) *trachomatis*, *Mycoplasma* (*M.*) *genitalium* and *Neisseria gonorrhoeae* is thought to ascend into the upper reproductive tract causing inflammation [1,4, 12]. However, STIs and/or BV do not always result in PID, and it is not known which cases will progress to PID and what factors contribute to progression. The possibility of specific pathogenic strains, a combination of micro-organisms or a specific host immune response has all been postulated as possible contributing factors in the progression to PID, and/or as potential biomarkers (reviewed, [13]). Potentially, it is a combination of micro-organisms and their high absolute burden that is involved, as quantitative PCR detection of *C. trachomatis* and some BV-associated micro-organisms detected 87% of endometritis cases [13]. A recent study identified that a cervicovaginal microbiome consistent with a specific BV sub-type profile was associated with the risk of acquisition of *Chlamydia* [14]. BV-associated bacteria such as *Gardnerella vaginalis*, *Mycoplasma hominis*, *Prevotella*, *Fannyhessea* (previously *Atopobium*), *Megasphaera*, *Sneathia*, *Eggerthella*-like bacteria and other Gram-negative rods have been associated with increased risk of developing PID independently of STI status [15], and during co-infection with *Chlamydia* [16]. Conversely, in cell culture models, certain species of lactobacilli have been demonstrated to mechanistically protect against colonization, growth and adverse cellular responses [17,18]. PID is also associated with sexual activity, such as a higher number of sexual partners, and recently, it has been demonstrated that BV is sexually transmitted between heterosexual couples [19].

A eubiotic cervicovaginal microbiome is typically dominated by *Lactobacillus* species, and generally, *Lactobacillus* (*L.*) *crispatus* has been attributed with the most protective functions (reviewed, [20]). The vaginal microbiome can be classified into (at least) five community state types (CSTs), broadly differentiated by the presence or absence of a dominant *Lactobacillus* species [21]. The microbiome profile is associated with different susceptibility to urogenital diseases, with CST I (a dominance of *L. crispatus*) associated with providing the most protection from disease [22]. This beneficial role is attributed to the production of d-lactic acid and other metabolites by *Lactobacillus* which maintains a low pH, modulates host cell activities and limits pathogen colonization [23,25]. Importantly, the proportional abundance and which *Lactobacillus* is abundant is important, as *L. crispatus* is associated with the most beneficial profile of activity (produces the more beneficial d-lactic acid and produces other beneficial factors, such as bacteriocins) [23,25], whereas *L. iners* (reported as most abundant in CSTIII) has been frequently associated with STI-positive cohorts and produces more l-lactic acid and is generally less beneficial [23,25]. CST IV has no dominant lactobacilli and typically is dominated by a mix of micro-organisms, including a high abundance of BV-associated organisms, and is associated with an increased risk of STI [26,28].

Additionally, there is a known interplay between the microbiome and host immune response in the reproductive tract, but little is known about local tissue gene expression during PID. A number of studies have examined the immune responses occurring in participants with PID by examining blood studies [29,32], but these results may not represent the reproductive tract responses that are found during PID. Therefore, to better understand PID, the aim of this work was to examine the cervicovaginal microbiota species, meta-genomes, whole-genome sequences of isolates, antibiotic resistance profiles and host immune responses associated with PID.

## Methods

### Study design, participant recruitment and sample collection

**Fig. 1.**
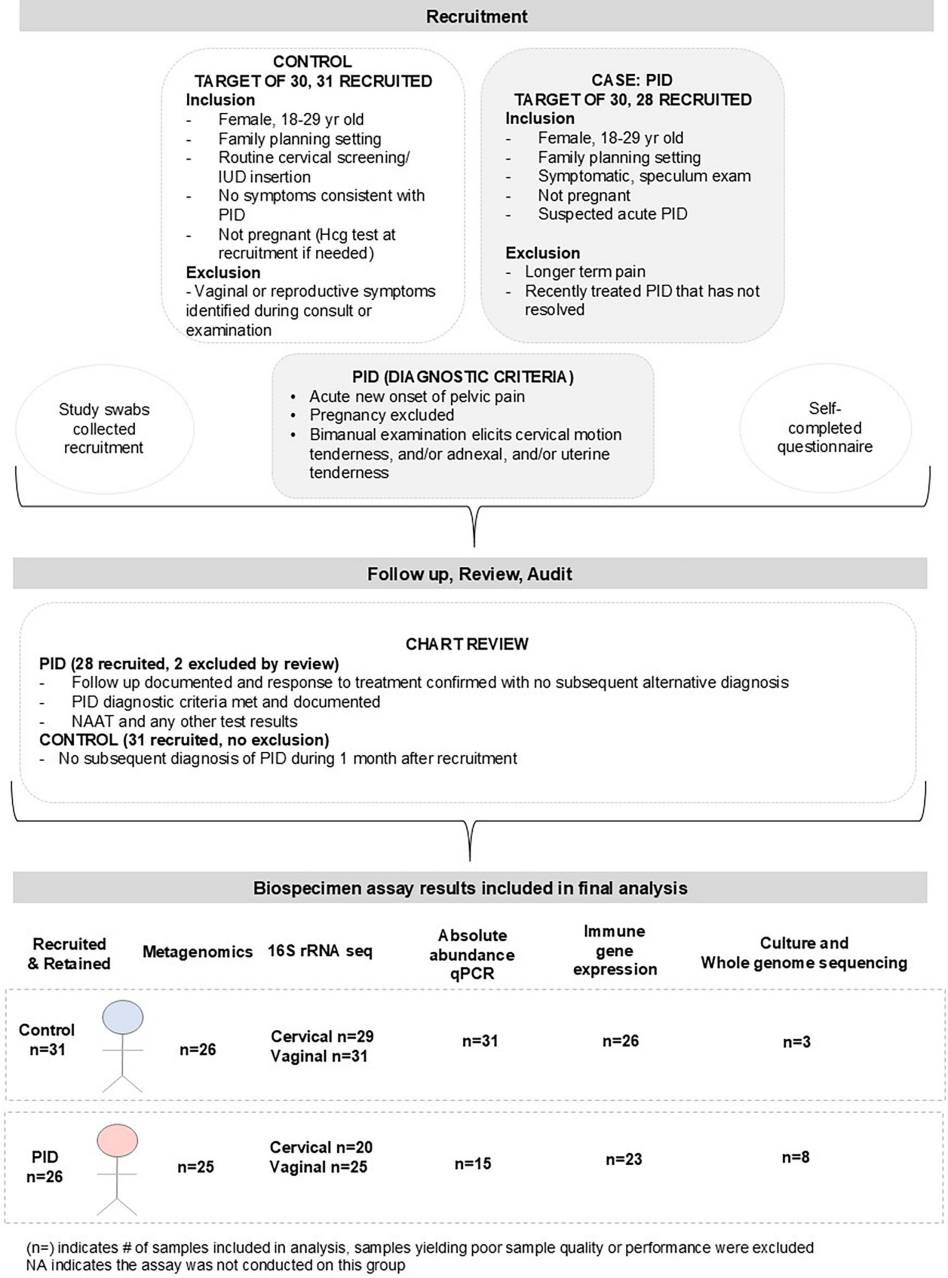
Schema of the PID case–control study design. The figure depicts the recruitment and inclusion/exclusion process of the clinical arm of the study and the number of samples included in the final biospecimen analysis and results shown throughout the paper. The recruitment inclusion and exclusion criteria are listed at the top; the follow-up, review and audit process in the middle; and the lowest component of the figure documents the samples that were at sufficient quality in extraction or results to be included in the analysis.

This was a case–control study, with cases recruited from a family planning clinic on presentation with signs and symptoms suggestive of PID, while controls were recruited from asymptomatic women presenting to the same service for routine cervical screening and/or IUD (intrauterine device) insertion procedures (Fig. 1). We aimed to recruit 60 participants consisting of 30 cases and 30 controls [33]. The sample size was estimated based on identifying a difference in the proportion of CSTs between the case–control groups with the assumptions of a 15% proportion in the controls and a 60% proportion in the cases, with 80% power and 5% significance. This would have required 19 participants in each group (a total of 38) [33]. Women presenting with symptoms suggestive of PID were recruited as cases and were retained in the study following a subsequent clinical chart audit when at least three members of the research team confirmed that the PID diagnostic threshold had been met. The PID diagnostic threshold was informed by the criteria outlined in the European Guideline for the Management of PID and the Australian STI Management guidelines to ensure diagnostic consistency among cases [34] (Fig. 1). Any participants lost to follow-up and/or with no evidence to confirm response to treatment were excluded. Pathology test results and any repeat visits up to 1 month after the initial recruitment presentation were also reviewed to ensure that no other subsequent diagnosis was later made (and to assign PID aetiology), and this was also conducted for controls. Any PID cases that did not subsequently meet the diagnostic threshold for PID during this chart audit were noted, and these samples and data were excluded from study analysis. Participant medical records and diagnostic test results from pathology providers were reviewed retrospectively to categorize cases according to PID subtype (idiopathic, instrumentation-associated or STI-associated), to confirm that a response to PID treatment had occurred at follow-up and to confirm that no alternative diagnosis was subsequently made that would exclude PID (two participants' data and samples were excluded by this process). Controls were eligible for inclusion if they were asymptomatic and presenting for routine cervical screening or IUD insertion at the participating family planning clinics. Cases and controls were eligible if they were women aged 18 to 29 years old (although a participant at 37 years old was included and approved by the ethics committee), English speaking and with the capacity to provide informed consent. Women were excluded from both groups if a biochemical pregnancy was confirmed via urine human chorionic gonadotropin testing, if they had a gynaecological malignancy or if a recent previous diagnosis of PID was unresolved (<3 months). Cases and controls completed a self-reported questionnaire regarding their medical, gynaecological, obstetric and sexual health history (Table S1, available in the online Supplementary Material). For both cases and controls, the participant samples, questionnaires and data sets were de-identified at the clinic. A total of 26 PID cases were eligible and included, and a total of 31 controls were included in the case–control analysis.

Cases underwent a speculum and bimanual pelvic examination to confirm the clinical diagnostic features of PID. During this procedure, routine swabs were collected according to the national guidelines[9], with three additional endocervical swabs and one high vaginal swab collected for the study. Controls underwent a speculum examination but did not have a bimanual examination unless clinically indicated, with collection of three endocervical swabs and one high vaginal swab for the study after any clinically indicated specimens (e.g. cervical screening swabs). The high vaginal and two endocervical swabs were collected into DNA/RNA Protect Cell Reagent (Qiagen), and one endocervical swab was collected into SPG buffer. Swabs were stored overnight in the clinic fridge (4 °C) and then shipped to the laboratory on dry ice before storage at −80 °C. Swabs for the case–controls were processed in batches with extraction and positive controls every 2 months.

### DNA extraction, shotgun metagenomics and 16S rRNA gene amplicon sequencing

Swabs collected and stored in DNA/RNA Protect Cell Reagent (Qiagen) were used for DNA and RNA extraction. DNA was extracted using enzymatic and mechanical lysis as described in Wee *et al*. [35]. Extraction controls and microbiota standards prepared using a mix of previously characterized samples with known CST (pooled samples from study [35]) were analysed with every sequencing run [36]. For shotgun metagenomics, the sequencing library was prepared by Macrogen Oceania, Australia, using the Nextera XT DNA Library Preparation Kit and sequenced on a NovaSeq 6000 (Illumina) (2×150 bp). For 16S rRNA, 2×300 bp amplicon sequencing of the 16S rRNA V3–V4 region was performed by the Ramaciotti Centre for Genomics (Australia) using 341F and 805R primers on the Illumina MiSeq platform [37]. Accession numbers, sequencing and post-quality-filtered read statistics are provided in Tables S2 and S3.

### Shotgun metagenomics data analysis

MetaWRAP-Read_qc module (v1.3.2) was used to trim and filter out reads that mapped to the human genome (Hg38). The resulting clean reads were used for taxonomic and functional profiling using MetaPhlAn4 (v4.0.6) (mpa_vOct22_CHOCOPhlAnSGB_202212 database) and HUMANn3 (v3.7) (v201901_v31 database) and assembly of metagenome-assembled genomes (MAGs) using MetaWRAP (v1.3.2) as previously described [38]. High- and medium-quality MAGs with a minimum completeness >70% and contamination <5% were kept. The taxonomy identity of each MAG was assigned using PhylophlAn3 (v3.0.3) and the reference SGB.Dec19 database [39]. MAGs with a mash distance of <5% were considered to belong to the same species [40]. For novel, uncharacterized MAGs, microbial traits (e.g. Gram reaction and associated morphology and atmospheric requirements) were predicted using Traitar (v1.1.2) [41]. Merged MetaphlAn4 table was used for alpha and beta diversity analysis.

Hierarchical clustering analysis (HCA) heatmaps were performed using Bray–Curtis distance to determine if there were any clustering of samples, using hclust2 ([41]v1.0.0), and to assign CSTs. To visualize and evaluate if the observed clusters in HCA were supported, a non-metric multidimensional scaling (NMDS) plot was created in Primer-e v6. In Primer-e, the relative abundance data from MetaphlAn4 were square-root normalized, and a resemblance matrix using Bray–Curtis distance was created. On the NMDS plot, Spearman correlation analysis of taxa was then overlayed using a cutoff value of >70% to identify species that were highly correlated with CSTs. CST assignment based on MetaPhlAn4 HCA was also compared with CST assignment from mgCST-classifier using the VIRGO1 database [42,43]. mgCST-classifier classifies samples into 27 mgCST clusters which were collapsed into the 5 main CSTs based on dominance/lack of dominance of canonical *Lactobacillus* species (CST I – mgCST 1–6, CST II – mgCST 7–9, CST III – mgCST 10–14, CST IV – mgCST 17–27 and CST V – mgCST 15–16).

### 16S rRNA gene sequencing analysis

16S rRNA gene sequence data were analysed using the Qiime2 platform (v2023.5.1) [44]. Reads were first trimmed using cutadapt, merged using vsearch and then denoised with Deblur. A phylogenetic tree was created using q2-phylogeny. Representative sequences from Deblur were then exported to STIRRUPS (Species-level Taxon Identification of rDNA Reads using Usearch Pipeline Strategy) for species-level classification of OTUs using an identity threshold of 97% and the vaginal 16S V1–V3 rDNA reference database [45]. The feature table, taxonomic table, unrooted tree, representative sequences and metadata table were all imported into the Phyloseq R package (v1.44.0) and merged [46]. Taxonomy was collapsed to the species level, and samples with less than 4,000 reads were filtered, rarefied to 5,000 reads, and a count table was produced. HCA using Bray–Curtis distance was then performed with hclust2 (v1.0.0).

### Isolation of lactobacilli isolates, whole-genome sequencing and antibiotic susceptibility testing

Participant swabs (endocervical) collected in 2-SP buffer [47] were streaked onto *Lactobacillus*-selective Man–Rogosa–Sharpe (MRS) agar and cultured anaerobically at 37 °C. Single colonies were then isolated and inoculated onto a new MRS agar (as above) to obtain pure cultured isolates. These isolates were then used for genomic extraction and characterized by Sanger sequencing of the 16S rRNA V1–V3 region. In total, 11 *Lactobacillus* isolates were recovered (Table 1) and characterized using whole-genome sequencing and antibiotic susceptibility testing. For whole-genome sequencing, *Lactobacillus* genomic DNA was isolated from cultures, and Illumina DNA libraries were prepared using Nextera XT DNA library Preparation Kit and sequenced using 2×150 bp chemistry. The reads were then trimmed using bbduk, and genomes were assembled using SPAdes (v3.15.5) with default settings.

**Table 1.** Clinical and demographic factors of participants enrolled in this study

Participants		Case	Control	Case vs control
**n=(%)**		26	31	*P*-value
**Age†**	**Median (range)**	21.5 (16–37)	25 (19–29)	0.008*
**PID aetiology**				
Idiopathic		16	na	-
Instrumentation		3	-	-
STI		7	-	-
**STI diagnosis confirmed from study entry**				
Number and % STI Pos		7 (26.9%)	0	-
**Which STI**				
Ng		0	-	-
Mg		5	-	-
Ct		4	-	-
†2 participants had both Ct and Mg				
**Contraceptive current‡**				
Number (% of responses)	**None**	3 (11.5)	2 (6.6)	0.655
	**Implanon**	4 (15.4)	1 (3.3)	0.172
	**Oral contraceptive pill**	6 (23)	8 (26.6)	>0.999
	**Hormonal IUD**	7 (29.9)	2 (23.3)	0.764
	**Copper IUD**	1 (3.8)	2 (6.6)	>0.999
	**Condom**	3 (11.5)	10 (33.3)	0.111
	**Vaginal ring**	0	1 (3.3)	-
	**Depo Provera**	1 (3.8)	0	-
	**Withdrawal**	0	2 (6.6)	-
	**Mini-pill**	0	2 (6.6)	-
	**Other**	1 (3.8)	0	-
Number of responses		**26/26**	30/31	-
**History of vaginal symptoms**				
Number (% of responses)	**Within the last 12** **months**	18 (16.9)	9 (30)	0.006*
Number of responses				
**Antibiotic use within the past 3 months‡**				
Number (% of responses)		10 (40)	4 (13.8)	0.030*
Number of responses		25/26	29/31	-
**Previous STI‡**				
Number (% of responses)	***Chlamydia***	13 (50)	6 (20)	0.048*
	**Gonorrhoea**	2 (7.7)	0	0.204
Number of responses		26/26	30/31	-
**Fertility‡**				
Number (% of responses)	**Past pregnancy**	8 (23)	9 (34.6)	0.541
Number of responses		26/26	26/31	-
**Sexual history‡**				
Number of Partners past 3 months	0	1 (3.84)	2 (6.45)	0.048*
Number (% of responses)	1	13 (50)	20 (64.5)	
	2–4	8 (30.79)	3 (9.68)	
	>5	2 (7.69)	1 (3.23)	
Number of responses		24/26	26/31	-
Number of Partners lifetime	0	1 (3.84)	1 (3.23)	0.027*
Number (% of responses)	1	7 (26.92)	16 (51.6)	
	2–4	12 (46.15)	5 (16.1)	
	5–7	2 (7.02)	3 (9.68)	
	>7	2 (7.92)	1 (3.23)	
**Number of responses**		24/26	26/31	-

*Indicates significant *P*-value (*P*<0.05); % shown on the table indicates the % of the respondents who answered that specific question or for whom the data were available, rather than the whole group. IUD refers to an intrauterine device. STI diagnosis was conducted by the pathology provider used for that clinic using the test routinely used in that setting (NAAT), and recorded pathology results were then recorded during chart audit for this study to note the STI test result.

†Mann–Whitney U test (*P*<0.05).

‡Fisher’s exact test (*P*<0.05).

For antibiotic susceptibility indications, pure cultures of *L. iners*, isolated as described above, were resuspended in 200 µl of peptone. From this mixture, 150 µl was inoculated onto Columbia Horse Blood agar. For *L. crispatus*, cells were also resuspended in 200 µl of peptone. Twenty-five microlitres of this mixture were then mixed with 5 ml of MRS soft agar and overlayed onto 15 ml of MRS base agar. Azithromycin (AZM; 15 mcg), DOX (30 mcg) and amoxicillin (AML; 10 mcg) antibiotic discs were then added to each plate. The plates were incubated anaerobically for 2 days at 37 °C after which the zone of inhibition (ZOI) was measured. This was used to provide a difference in susceptibility to the antibiotics in the isolates selected and cultured here, rather than a quantitative resistance profile.

### Phylogenetic analysis of MAGs and pure genomes from selected species

Phylogenetic analysis of MAGs belonging to *L. crispatus*, *L. iners* and *G. vaginalis* was performed to determine if the strains found in cases were different from controls. For *L. crispatus* and *L. iners*, assembled genomes from *Lactobacillus s*trains isolated from pure culture from the same samples were included. A maximum parsimony tree for each species was generated by kSNP4 using a ‘k-mer’ size of 17, as this was the optimal k-mer length predicted by the Kchooser4 command in kSNP4 [48]. The phylogenetic tree output was visualized using iTOL (v6.9).

### Prediction of antimicrobial resistance in species

To identify the presence of antimicrobial resistance (AMR) genes and point mutations in assembled MAGs from cases and controls, the abritAMR pipeline (version 1.0.14) was used with default commands [49,50].

### Quantification of selected bacteria using a calibrated real-time PCR assay to provide a copy number estimate

Species-specific quantitative PCR was conducted to provide a quantitative assessment of abundance data [calibrated copy estimates by quantitative real-time PCR (qPCR)]. Previously described primers targeting the 16S rRNA gene sequence for *L. crispatus*, *L. iners*, *Fannyhessea vaginae* (previously *Atopobium vaginae*), *G. vaginalis*, *Prevotella* species and *M. genitalium* were used (Table S4) [51,52]. Standard curves were created using synthetic DNA (Integrated DNA Technologies) which consisted of the target amplicon with an additional 15–20 bases (Table S5). Each qPCR reaction contained 12.5 µl AmpliTaq Gold 360, 0.5 µM F/R primers, 5 µl EVA green, 5 µl DNA and 0.5 µl H_2_O. qPCR cycling was performed in the Qiagen Rotor-Gene Q-5plex platform with the following cycling conditions: 95 °C for 10 min followed by 40 cycles of 95 °C for 15 s and 60 °C for 60 s.

### RNA extraction and RT-qPCR

RNA was extracted from endocervical swabs collected in RNAProtect using the Qiagen RNeasy Plus Mini Kit and protocol. Of the 57 case–control samples, 49 samples (23 cases, 26 controls) were extracted for sufficient quality for RT-qPCR analysis of 84 innate and adaptive immune-associated genes (Fig. 1). cDNA was synthesized, and RT-qPCR was performed using the Qiagen RT^2^ profiler array (96-well format) for human innate and adaptive immune responses following the manufacturer’s instructions (Cat. No. 330231, PAHS-052ZA). The comparative threshold cycle (C_t_) method (2^-ΔΔCt^) was used to determine relative gene expression [53].

### Preliminary biomarker prediction and analysis

Receiver operating characteristic (ROC) analysis was performed to assess the sensitivity and specificity of significant species identified in Linear Discriminant Analysis (LDA) effect size (LEfSe) analysis and for differentially expressed immune genes as potential biomarkers for PID. This analysis was not considered in the original sample size plan for the study and should be interpreted as a preliminary analysis to determine potential targets for future analysis given the modest sample size and lack of an independent validation cohort or group conducted in this study.

### Statistical analysis

The statistical analysis was conducted in GraphPad Prism version 10, or in the appropriate bioinformatics suite in which the analysis was implemented as outlined in the methods sections above. Each result figure and methods section indicates which statistical method was implemented for any analysis where a *P*-value is provided and the number of samples analysed. The clinical and demographic data were analysed using IBM SPSS Statistics (Version 30).

For metagenomics, alpha diversity metrics (species richness, Shannon’s diversity, Simpson’s diversity and Gini’s diversity) were graphed using GraphPad Prism (v10), and statistical analysis was performed using the Mann–Whitney U test. For beta diversity, an analysis of similarity (ANOSIM) was performed to see if there was significant separation between clusters with random permutations set at 9,999 and a universal Bonferroni correction applied for multiple pairwise testing [1,2, 54]. CST assignment agreement between 16S and metagenomics was assessed using unweighted Cohen’s kappa co-efficient in GraphPad online calculator (https://www.graphpad.com/quickcalcs/kappa2/).

Significant taxa (at the species and genus levels) and microbial metabolic pathways enriched in PID and controls were first identified using LEfSe (version 1.0) analysis on the Galaxy platform [55]. A *P*-value<0.05 and log10 LDA score >3 were used as cutoffs. Significant taxa identified using LEfSe were then confirmed using ANCOM-BC. Estimated read counts from MetaphlAn4 were obtained using the option -t rel_ab_w_read_stats and imported into the Phyloseq R package (v1.44.0). ANCOM-BC in the microbiomeMarker R package (1.13.2) was performed with default parameters, except that taxa prevalence was set at >5% (prv_cutoff=0.05), and ‘fdr’ was used as the *P*-value correction method. A *P*-adjusted value <0.05 was considered significant. In the ROC analysis, the optimal cutoff values for each single biomarker were obtained using the pROC R package [56], and multiple testing correction was performed using the Benjamini–Hochberg method. CombiROC [57] was used to evaluate the diagnostic potential for multiple combined biomarkers, with a positive detection threshold of 0.01 set, and only combinations with an area under the curve (AUC) >0.8 were selected. The qPCR analysis for immune gene expression was analysed using the ΔΔct method, and a Student’s t-test was used to determine statistical significance. Multiple testing correction was performed using the Storey and Tibshirani false discovery rate method. qPCR analysis of bacterial copy number was analysed using the Mann–Whitney U test for each gene, and Benjamini–Hochberg to correct for multiple testing. In the case of assembled MAGS, a Fisher’s exact test was used to test if species or participant group had a higher representation of AMR-associated genetic features.

## Results

### PID cases were strongly associated with the presence and estimated copy number abundance of *G. vaginalis* and other CST IV CST micro-organisms, regardless of aetiology

We implemented a case–control approach to enable us to compare cervicovaginal microbial and immune factors between PID cases and healthy asymptomatic controls at participating Family Planning Clinics in NSW, Australia. The flowchart in Fig. 1 describes the recruitment and study sample inclusion process. The clinical chart audit and self-reported questionnaire data were analysed to understand the demographics, clinical features and risk factors of both groups. The PID cases had demographic and behavioural factors consistent with those previously associated with PID (Table 1) [58]. These included the following: PID cases were significantly younger (*P*-value=0.008) and had a higher proportion of self-reported recent history of vaginal symptoms (16.9% versus 30%, *P*-value=0.006) (Table 1). A history of chlamydia was found to be more frequent in cases (50%) than in controls (20%) (*P*-value=0.048), while in this study, a history of gonorrhoea was not significantly different. A higher number of sexual partners was more frequently reported by the PID cases (*P*-value=0.048 and 0.027, respectively, for the past 3 and 12 months). Self-reported use of antibiotics within the preceding 3 months was more common in PID cases (40%) compared to controls (13.8%) (*P*-value=0.030) (Table 1). In the cases, there were 61.5% idiopathic, 11.5% instrumentation and 26.9% STI-associated aetiologies (Table 1). The use of different contraceptives and history of past pregnancy did not differ between the case and control groups.

**Fig. 2.**
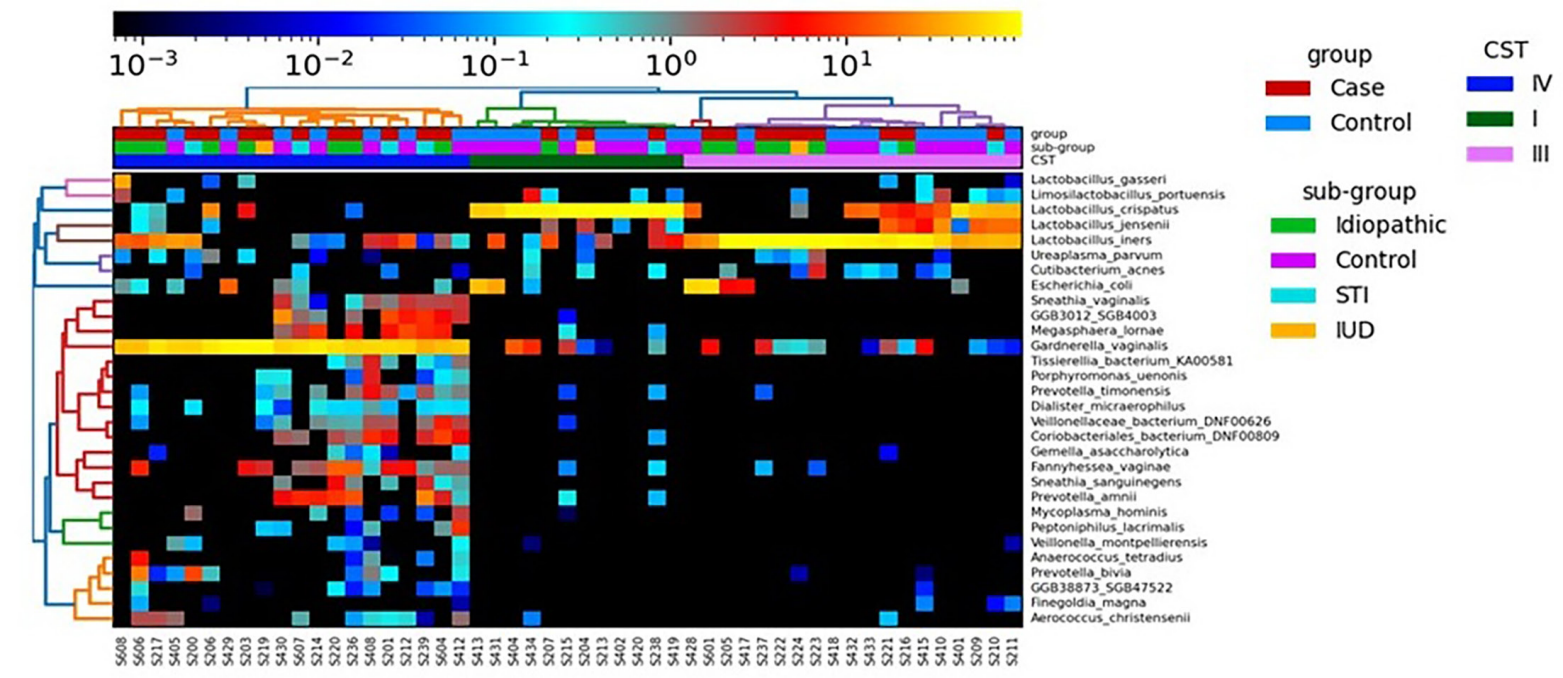
Hierarchical cluster dendrogram heatmap of the relative abundance of the top 30 species identified using metaphlAn4. Sample IDs are on the bottom (x-axis), and the species are on the right (y-axis). Group, metadata and assigned CST are shown on the top with the legend on the bottom. The heatmap for all species is in Fig. S1.

**Fig. 3.**
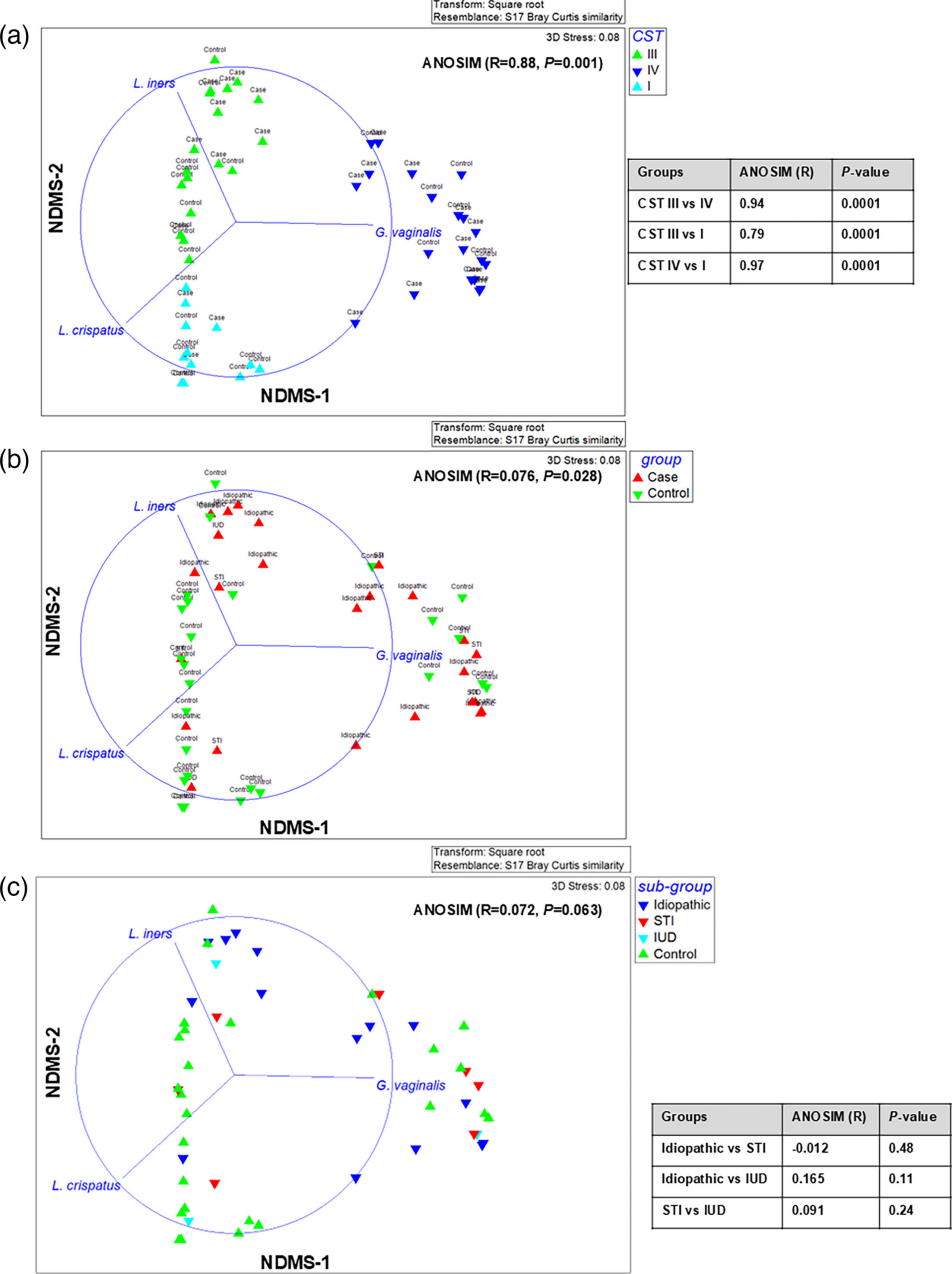
Taxa and microbial features from metagenomics analysis associated with PID. Three-dimensional NMDS plot generated from a Bray–Curtis resemblance matrix and coloured by (**a**) CSTs as assigned in dendrogram heatmap, (**b**) group (case and control) and (**c**) sub-groups from the case and control groups (control, idiopathic, STI and IUD). The annotated labels are shown on the right of the NMDS plot. The table shows pairwise ANOSIM comparison between individual CSTs and sub-groups with *P*-value significant cutoff determined using a universal Bonferroni correction (cutoff *P*=0.0167). Significant pairwise *P*-value is indicated with *. Species with a Spearman correlation >0.7 are overlayed onto the plot.

A series of shotgun metagenomics, 16S rRNA gene amplicon and species-specific qPCR protocols were conducted on genomic DNA from the collected bio-specimens to determine and analyse the microbiome and microbial features. An HCA of the shotgun metagenomes was used to determine if there was any clustering between the cases and controls (Figs 2 and S1). The cervical microbiome of participants clustered into three groups here, with two groups (CST II and V) previously reported as not detected in this study [21]. This is the first report using metagenomics for PID. One cluster contained a group of heterogeneous anaerobic species, with *G. vaginalis* often being the dominant species and hence assigned as CST IV. One cluster was dominated by * L. crispatus* (assigned CST I). A third cluster was dominated by *L. iners* (assigned CST III) (Fig. 3a). The relative abundance of canonical dominant *Lactobacillus* spp. and *G. vaginalis* within each CST is shown in Table S6. The separation of these three clusters was supported by an ANOSIM test, which showed a large and significant effect size between each cluster, driven by the species indicated on the figure (R=0.88, *P*-value=0.001, Spearman Rho’s>70%) (Fig. 3a). Spearman correlation of *L. crispatus*, *L. iners* and *G. vaginalis* supported the assignment of CST I, III and IV, respectively, in the metagenomics cluster (Spearman Rho’s>70%) (Fig. 3a). Only one sample (S608) differed in CST assignment between the technologies; this sample was assigned as CST II by 16S but CST IV using metagenomics. The NMDS plot showed that PID tended to group towards the top right side with CST III/IV, while controls tended to group in the bottom left with CST I (Fig. 3b). This separation was confirmed with an ANOSIM test showing a separation between cases and controls (ANOSIM R-value=0.076 and *P*-value=0.028) (Fig. 3b). A further Chi-squared test also found a trend towards significance for an association between PID and CST (*P*=0.08). No separation between PID subgroups based on aetiology was observed (Figs 3c and S1). To further confirm our findings, a second cervicovaginal microbiome-specific database (VIRGO) and classification method (mgCST-classifier) were performed which identified 12 out of the 27 possible mgCST clusters in our cohort (Fig. S2). When collapsed into the five main CSTs, mgCST-classifier main CST assignments showed substantial agreement with our initial MetaphlAn4 CST assignment (κ=0.736). A trend towards significance was also found between PID and mgCST association (*P*=0.0582) which was consistent with PID and MetaphlAn4 CST association (*P*=0.08). Alpha diversity for the shotgun metagenomics data from the cervix was evaluated to examine the relative species abundance and diversity within each sample (Fig. S3). No significant differences were observed, indicating that the overall number of species and their relative abundance were similar between cases and controls.

16S rRNA gene amplicon sequence analysis showed that the relative abundance of microbial species between the cervix and vagina was largely concordant for individuals (Fig. S4) with substantial agreement for CST assignment (κ=0.761). Cohen’s kappa co-efficient also showed moderate to substantial agreement for CST assignment between shotgun metagenomics and cervical/vaginal 16S rRNA amplicon sequencing (κ=0.551–0.732). Like the findings using metagenomic approaches, CST IV was significantly associated with PID cases (chi-squared test, *P*=0.0331 and *P*=0.0232 for cervical and vaginal 16S rRNA, respectively) (Figs S5A and S4B).

**Fig. 4.**
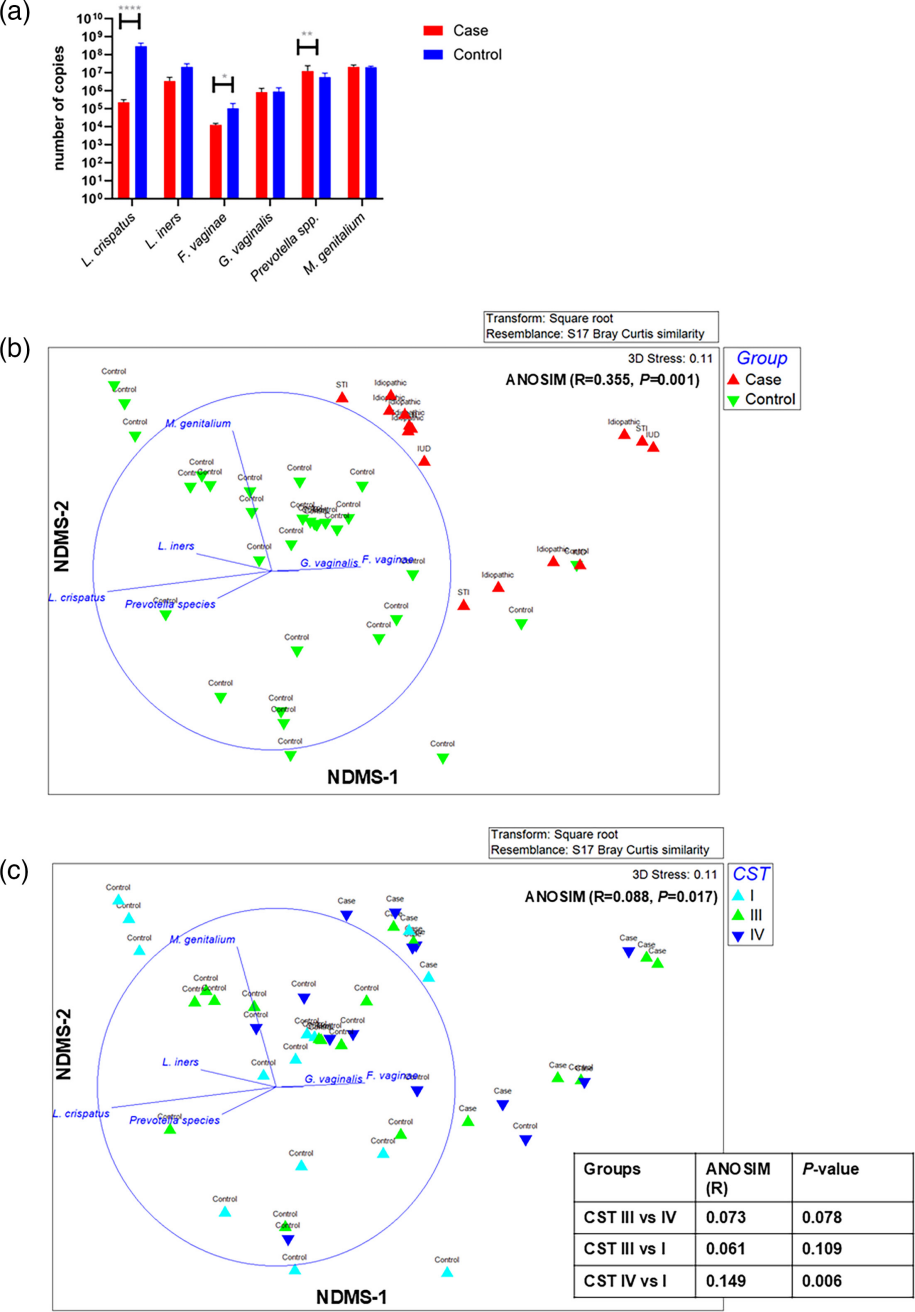
Abundance of cervicovaginal microbes in PID. (**a**) qPCR to establish quantities of selected bacterial species. Cases are indicated by red circles, and controls by blue boxes. The y-axis is copy number (of target gene) per sample using calibrated copy number abundance determined with a standard curve. The x-axis indicates each species or genus tested by the qPCR. Mean is indicated by the box, and sd is shown as indicated by the lines. Significance was calculated using the non-parametric Mann–Whitney test for each gene by case–control status. ****: Benjamini–Hochberg *P*-adjusted value<0.0001; **: *P*-adjusted value<0.01; *: *P*-adjusted value<0.05. (**b**) Three-dimensional NMDS plot generated from a Bray–Curtis resemblance matrix using calibrated copy estimate data (qPCR data) and coloured by (**b**) group (case and control) and (**c**) CST. The Spearman correlation of each taxon is shown on the plot, and the table shows pairwise ANOSIM comparison with the *P*-value significant cutoff determined using a universal Bonferroni correction (cutoff *P*=0.0167). Significant pairwise *P*-value is indicated with *.

Species-specific qPCR was performed on cervical sample DNA extractions relative to standard controls to quantify the calibrated estimates of the copy number to show the abundance of selected species. *L. crispatus* was found in higher estimated abundance in cervical samples from women in the control compared to case (*P*-adjusted=0.0006) (as did *F. vaginae* to a lesser extent) (Fig. 4a). *Prevotella* spp*.* had higher copies in the cases compared to controls (Fig. 4a; *P*-adjusted values=0.0792 and 0.0075, respectively). Importantly, no differences were identified for *L. iners*, *G. vaginalis* and *M. genitalium* between case and control (Fig. 4a). However, combining all 6 of these taxa (estimated abundance using a calibration curve to measure copy numbers) using an NMDS plot showed greater separation between PID cases versus controls and had a larger effect size (ANOSIM R-value=0.355 and *P*-value=0.001) (Fig. 4b) compared to relative abundance analysis. The separation of CSTs (assigned using metagenomics) was relatively poor with only CST IV vs CST I found to be significant (Fig. 4c). The estimated copy number abundance of these six taxa did not significantly differentiate subgroups of the PID aetiology (Fig. 4b). While the sample size is small, this could suggest that regardless of the presence of an STI, the estimated copy number abundance of some CST IV organisms and absence of * L. crispatus* may be an important contributor to PID.

### LEfSe identifies *Gardnerella* and *Fannyhessea* as being enriched in PID cases compared to controls which were enriched with *L. crispatus*

**Fig. 5.**
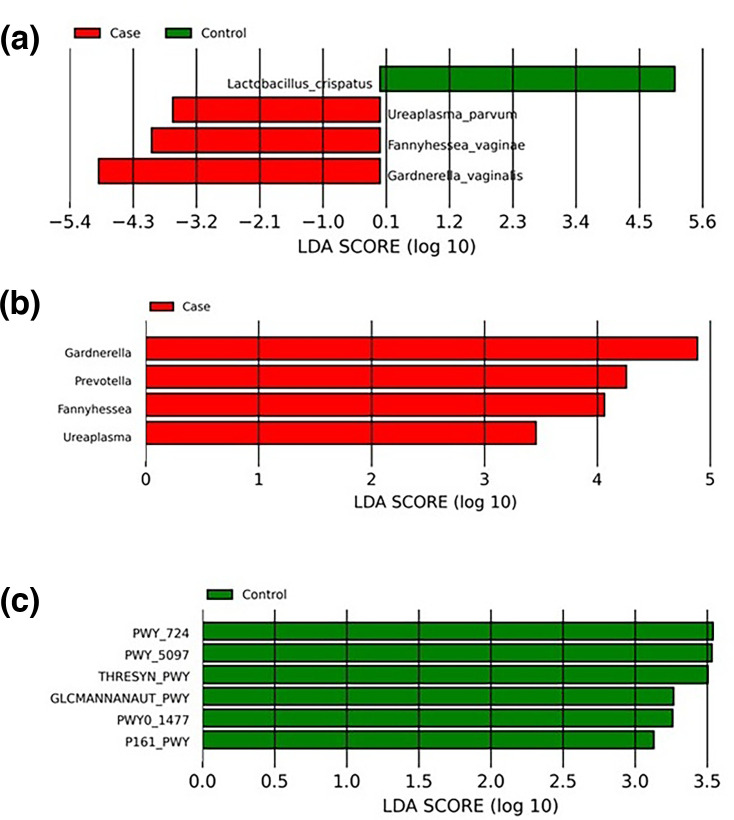
Metagenomic analysis of species and pathways associated with PID. (**a**) Histogram of significantly enriched taxa identified using LEfSe at the species level and (**b**) genus level from metagenomics analysis. (**c**) Shows enriched microbial metabolic pathways in controls. Pathway PWY_ 724 is super pathway of l-lysine, l-threonine and l-methionine biosynthesis; PWY-5097 is l-lysine biosynthesis VI; THRESYN-PWY is super pathway of l-threonine biosynthesis; GLCMANNANAUT-PWY is super pathway of *N*-acetylglucosamine, *N*-acetylmannosamine and *N*-acetylneuraminate degradation; PWY0-1477 is ethanolamine utilization; P161-PWY is acetylene degradation (anaerobic).

To determine if there were specific species enriched in PID, we performed LEfSe analysis on the metagenomic dataset and identified three species (*F. vaginae*, *G. vaginalis* and *Ureaplasma parvum*) that were significantly enriched in the cases compared to the controls. *L. crispatus* was the only species enriched in controls (Fig. 5a). At the genus level, LEfSe analysis showed that *Gardnerella*, *Fannyhessea* and *Ureaplasma* [3] were significantly enriched in cases with the addition of *Prevotella* also being significantly enriched [LDA score (log10)>|3|] (Fig. 5b). However, in controls, the *Lactobacillus* genus was not enriched, suggesting that only *L. crispatus* is associated with healthy controls. ANCOM-BC analysis at the species level found similar associations as LEfSe with *G. vaginalis* (W=−2.83, raw *P*-value=0.0047, p.adjust=0.052), *F. vaginae* (W=−1.93, *P*-value=0.053, p.adjust=0.38), *U. parvum* (W=−1.51, raw *P*-value=0.129, p.adjust=0.54) and *L. crispatus* (W=1.5, raw *P*-value=0.13, p.adjust=0.54). However, only *G. vaginalis* was borderline significant after multiple testing correction, possibly due to the relatively small sample size of our study. At the genus level, ANCOM-BC found no association to *Lactobacillus* (W=0.62, raw *P*-value=0.53, p.adjust=0.83), but *Gardnerella* (W=−2.94, raw *P*-value=0.003, p.adjust=0.064), *Fannyhessea* (W=−2.04, raw *P*-value=0.04, p.adjust=0.33), *Prevotella* (W=−2.27, raw *P*-value=0.023, p.adjust=0.23) and *Ureaplasma* (W=−1.73, raw *P*-value=0.08, p.adjust=0.48) showed the same trend in association as LEfSe, but only *Gardnerella* was borderline significant after multiple testing corrections.

The HUMAnN3 pipeline and LEfSe analysis identified significantly enriched microbial metabolic pathways associated with PID and controls. Six microbial pathways were significantly associated with controls (LDA score log10 >3). These pathways were involved in the super pathway of l-lysine, l-threonine and l-methionine biosynthesis (PWY-724); l-lysine biosynthesis VI (PWY-5097); super pathway of l-threonine biosynthesis (THRESYN-PWY); super pathway of *N*-acetylglucosamine, *N*-acetylmannosamine and *N*-acetylneuraminate degradation (GLCMANNANAUT-PWY); ethanolamine utilization (PWY0-1477); and anaerobic acetylene degradation (P161-PWY) (Fig. 5c). No microbial metabolic pathways were enriched in cases. The microbial species associated with metabolic pathways significantly enriched in controls were analysed. *L. crispatus* was found to be the main contributing species to all six pathways for most control samples (Figs S6A–S6F). The relative abundance of each pathway showed good separation between control and cases (Figs S6A–S6F).

### Phylogenetic analysis of MAGs indicates no strain differences between cases and controls

**Fig. 6.**
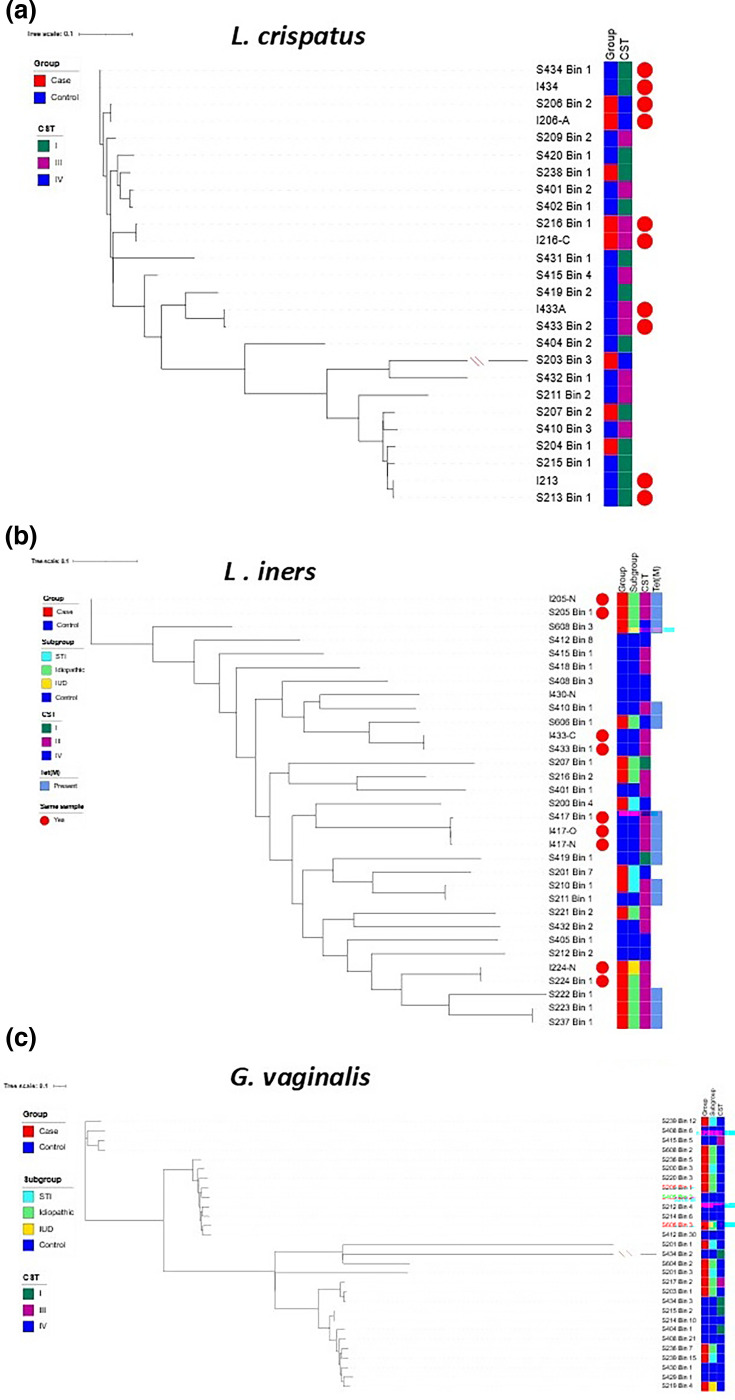
Maximum parsimony tree for key organisms. (a) *L. crispatus*, (b) *L. iners* and (c) *G. vaginalis*. Matching MAGs (named as ‘Bin’ beside sample code) and cultured isolate genomes (indicated by the ‘I’ at the front of the sample code) from the same samples are indicated by the red circle. The PID groups and CSTs are shown on the right. The tree is read from left to right. The PID group, CSTs and presence of Tet(M) are shown on the right.

To determine whether there are different strains found in cases and controls, MAGs were assembled and analysed. From the 51 samples sequenced (samples with insufficient DNA yield were excluded), 186 medium- to high-quality MAGs (>70% completeness, <5% contamination) were successfully assembled (Table S7). Of the 186 MAGs assembled, *G. vaginalis* was the most common, followed by *L. iners*, *L. crispatus* and *F. vaginae* (Fig. S7). This result is consistent with the MetaPhlAn4 results. Finally, four taxa of unclassified MAGs (GGB3012_SGB4003, GGB34027_SGB48293, GGB4807_SGB6646 and GGB1460_SGB2023) belonging to the *Hungateiclostridiaceae*, *Prochloraceae*, *Lachnospiraceae* and *Bacteroidales*_unclassified families were also identified (Fig. S7). Microbial phenotype prediction using Traitar identified SGB48293 and SGB2023 as strict anaerobic Gram-negative rods, while SGB6646 was a strict anaerobic Gram-positive rod. SGB4003 was also identified as Gram-positive, but its morphology and atmospheric growth conditions could not be determined (Fig. S8). All four were glucose fermenters. GGB4807_SGB6646 (*Lachnospiraceae*) MAGs from this study had high average nucleotide identity (ANI >98.5%) to publicly available *Candidatus Lachnocurva vaginae* genomes (taxon=699240; formerly known as BVAB1), suggesting that they belonged to the same species [59]. For species with >20 MAGs recovered (*L. crispatus*, *L. iners* and *G. vaginalis*), phylogenetic analysis was performed to see if there were any differences between cases and controls. For *L. crispatus* and *L. iners*, genomes from the selection of strains isolated and cultured from matched samples were also included (Fig. 6a, b). These genomes all tightly clustered with their respective MAGs [e.g. S213 bin 1 and I213 (isolate) in Fig. 6c], suggesting that the assembled MAGs are good representations of the corresponding strains and samples. There was no clustering of *L. crispatus* or *L. iners* or *G. vaginalis* MAGs by case or control group or CST (Fig. S9).

### Microbial MAGs and *L. iners* isolates from PID cases indicated resistance to tetracycline

**Fig. 7.**
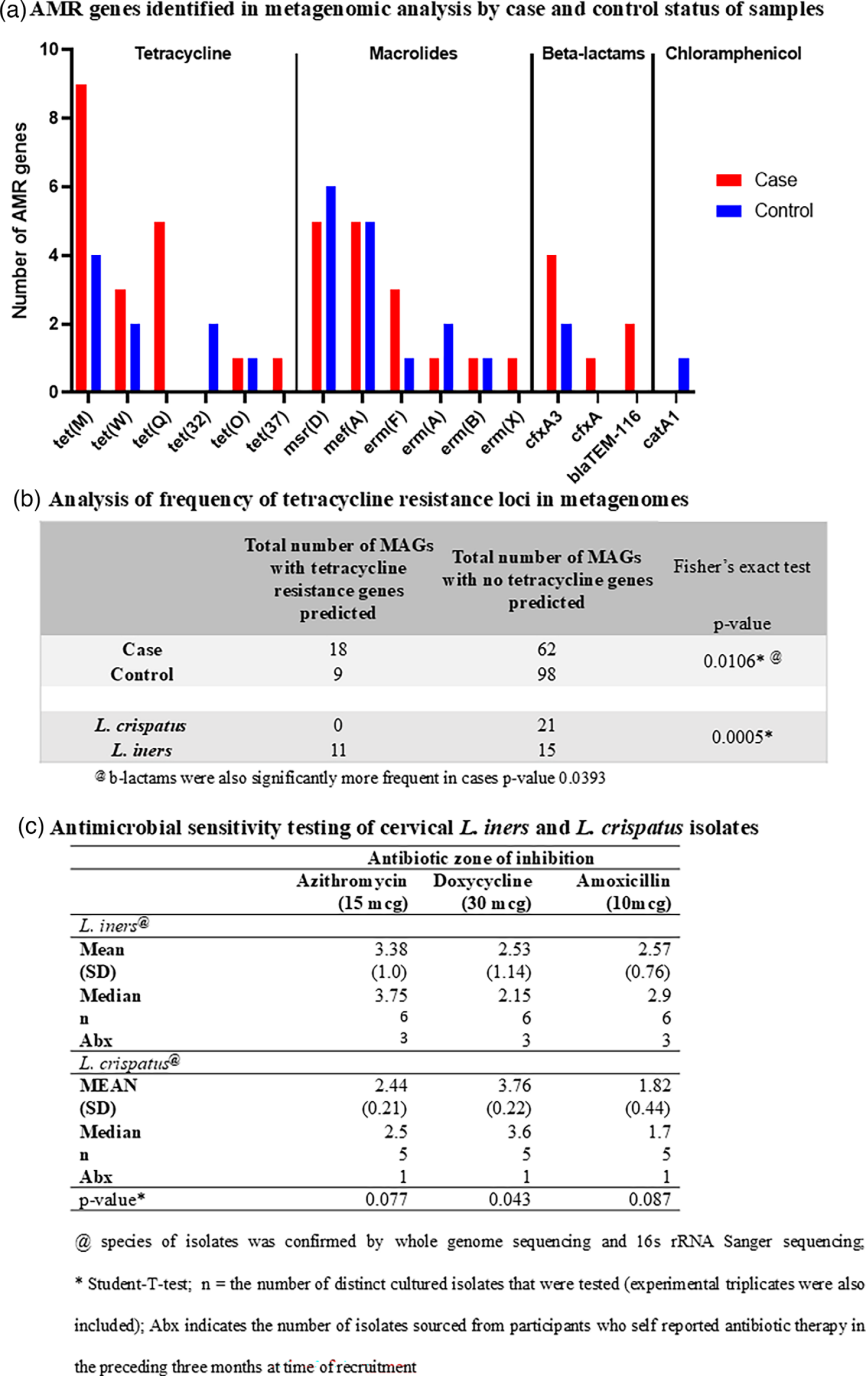
AMR is associated with PID and *L. iners*. (a) AMR resistance genes identified in the metagenomic analysis are indicated on the bar chart for four classes of antibiotics. The number of MAGs with predicted AMR resistance genes (y-axis), colour-coded by case (red) or control (blue) status and each locus identified (x-axis). (b) Analysis of frequency of tetracycline resistance loci predicted in MAGs. Tetracyclines are commonly used antibiotics, and DOX is recommended for chlamydial infections. Hence, the total tetracycline dataset in figure (a) was analysed in the context of the case–control, or species and frequency of predicted resistance. The figure shows the analysis. (c) *L. iners* and *L. crispatus* isolates cultured from participants in this study were tested for antibiotic susceptibility by measuring ZOI. The figure shows the zones of clearance and statistical testing for differences in susceptibility to the antibiotics listed at the top.

We examined if AMR profiles of organisms present in PID were distinct from controls using both genomics analysis and analysis of cultured isolates for susceptibility differences in a disc diffusion assay. The presence of AMR genes was assessed in both MAGs and whole-genome sequences from isolates. Resistance was frequently predicted for tetracyclines, followed by macrolides and then beta-lactams (Fig. 7a). Predicted resistance to tetracyclines was primarily conferred through the presence of *tet(M)*, *tet(Q)* and *tet(W)*, while for macrolides, it was through the *msr(D)*, *mef(A)* and *erm(F)*, *emr(A)* and *erm(B)* genes. The predicted AMR resistance across groups, CSTs and species was assessed. It was found that *L. iners* MAGs had a significantly higher number of AMR genes (largely tetracycline resistance genes) compared to *L. crispatus* (*P*=0.005). These were predominantly *tet(M)* (tetracycline resistance). PID cases also had a higher number of MAGs containing predicted tetracycline-resistant genes (*P*=0.0104) (Fig. 7b).

To confirm reduced susceptibility of *L. iners* to tetracycline, ZOI assays were performed for a selection of *L. iners* and * L. crispatus* isolates cultured from participant samples (Fig. 7c). Three *L. iners* genomes from isolated strains contained *tet(M)*, and the same samples corresponding to MAGs also contained *tet(M)*, validating the predicted resistance in metagenomics analysis. None of the cultivated and isolated *L. crispatus* genome sequences were predicted to encode *tet(M)*. Larger (but not significant) average ZOI for AZM and AML were observed for these *L. iners* isolates (3.38 and 2.57 cm) compared to * L. crispatus* (2.44 and 1.82 cm), regardless of whether the isolate came from case or control. The average ZOI for *L. crispatus* (3.76 cm) to DOX was significantly greater than *L. iners* (2.53 cm) (*P*-value=0.043).

### Gene expression analysis indicated that a possible Th2 response is more common in PID cases

**Fig. 8.**
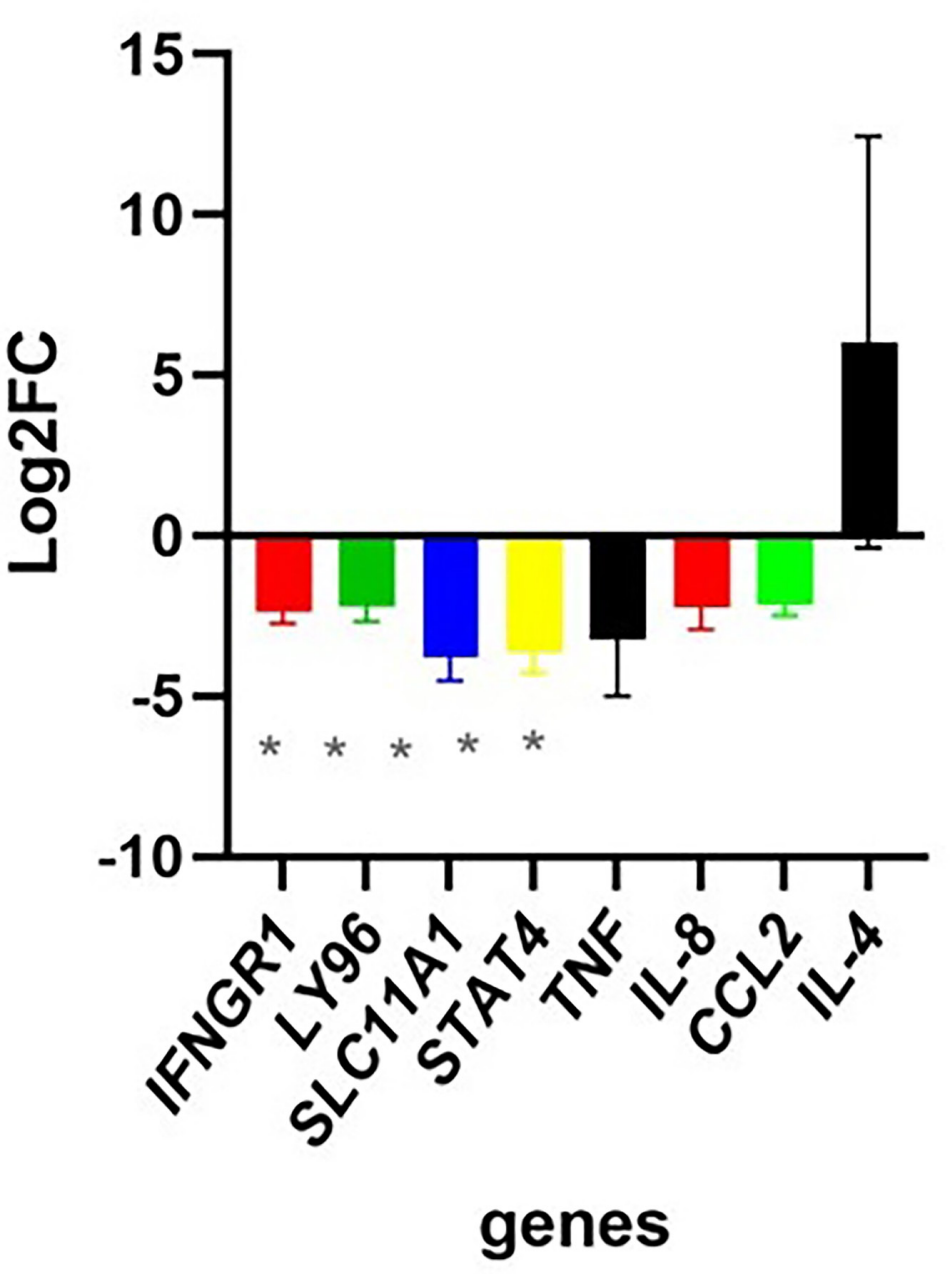
Immune-associated genes in PID. Histogram of differentially expressed immune-associated genes with significant differences detected between case and controls. On the x-axis is the gene name, and on the y-axis is the log2 fold change values. The asterisk (*) represents *P*<0.05, when a t-test for each factor was tested; these are shown in grey because when the analysis was adjusted for multiple factors, none remained significant.

RT-qPCR was performed to compare the expression of a selection of innate and adaptive immune-associated genes between PID cases and controls (Fig. S9). IFNGR1, LY96, SLC11A, STAT1 and TNF were significantly lower in expression in PID (*P*<0.05), whereas two additional genes, IL8 and CCL2, showed a trend towards lower levels (*P*-value 0.05 and 0.056, respectively) (Fig. 8). All seven of these genes are associated with a Th1 response and/or M1 polarization. IL-4, which is associated with Th2 response, was more highly expressed in PID cases compared to controls with a log2 fold change of 6; however, a Student’s t-test could not be performed, as expression of this gene was largely not detected in the controls (detected in 9 cases and 1 control).

### Preliminary analysis of microbiome and immune factors as possible biomarkers to detect PID

ROC curve analyses were performed to evaluate the sensitivity and specificity of using differentially expressed immune genes and the absolute and relative abundance of species to detect cases or controls. Single factors were identified (Fig. S10), and a combined microbiome biomarker panel was additionally assessed [57]. A combination of 4 species (*L. iners*, *L. crispatus*, *F. vaginae and U. parvum*) resulted in a high AUC (0.81) and was able to distinguish cases from controls (Fig. S11). This supports the notion that PID is likely due to a combination of organisms and further that the quantity of some organisms, as well as the proportional abundance of the organisms, could be associated with PID (or healthy controls in the case of *L. crispatus*). It should be noted that, typically, such analysis would include an independent validation group and a ‘gold standard’ diagnostic to compare. As such, these are presented as preliminary findings to support future hypothesis and sample size estimates for a larger-scale study.

## Discussion

The mechanism by which some women with STI or BV develop PID is not fully understood, and there are currently no molecular diagnostic tests for PID. The current clinical diagnostic methods are also subjective and invasive [8,60]. This study aimed to investigate the association between cervicovaginal microbiome and immune factors with PID, using a case–control study design, to identify contributing factors to disease and potential diagnostic biomarkers.

PID cases recruited for this study had clinical, sexual and demographic data that were consistent with previous studies (Hay *et al.*, 2016; Kreisel *et al.*, 2017; Simms *et al.*, 2006; Sweeney *et al.*, 2022). *L. crispatus* was associated with the vaginal and cervical microbiome composition on women in the control group in our study, both with proportional abundance and estimated quantity. *G. vaginalis*, *F. vaginae*, *U. parvum* and the *Prevotella* genus were associated with PID cases using a range of distinct analyses including estimated copy and proportional measures, and this was independent of the PID aetiology. The study has a small sample size, and this limitation should be noted; for example, the LEfSe analysis when adjusted for multiple testing showed fewer significant differences. Nonetheless, the observations here are consistent with previous literature. *G. vaginalis*, *F. vaginae* and *Prevotella* spp. are all BV-associated bacteria. *G. vaginalis* is one of the most frequently identified bacteria in BV [61]. Previous studies have found that BV and BV-associated bacteria were associated with increased risk of PID [62], consistent with our results. These studies and ours provide further evidence that PID is associated with dysbiosis of the genital microbiome and specifically a higher proportion and quantity (by copy number estimate) of up to six micro-organisms.

Shotgun metagenomic sequence analysis in this study detected known AMR genes conferring resistance to tetracycline and macrolides. Tetracycline-resistant genes were overrepresented in the MAGs sourced from the microbiome of PID cases and were detected in *L. iners* and some CST IV bacteria but not in *L. crispatus*. This is consistent with other studies which found higher rates of AMR genes and/or resistance to tetracycline and macrolides in women colonized with *L. iners* and CST IV bacteria [63,67]. We confirmed these observations *in vitro* on a subset of cultured isolates from the same women, where *L. iners* isolates displayed smaller ZOI when cultured with discs of tetracycline compared to *L. crispatus*. This has been observed in another context [18].

This study found that *L. crispatus*, but not *L. iners*, was associated with healthy controls. *L. crispatus* produces the highest amount of d-lactic acid, while *L. iners* produces l-lactic acid instead. d-lactic acid is more effective than l-lactic acid at inhibiting STI pathogens [17,68]. The vaginal levels of the lactic acid isomers have been shown to correlate with changed profiles of epithelial and defence-related responses, such as MMP-8, hyaluronidase-1 and other factors in the vagina, indicating a direct impact on the host defence mechanisms [69,70]. *L. crispatus* also contains additional genes for bacteriocins and pyruvate oxidase, which produces H_2_O_2_ [71,73], which also inhibit the colonization of harmful pathogens and are absent in *L. iners* [71]. We found six metabolic pathways enriched in controls, with *L. crispatus* the main contributing species to these pathways. These pathways are related to genetic differences between *L. crispatus* and *L. iners*. The l-lysine, l-methionine, l-threonine biosynthesis super pathway is unique in *L. crispatus* and was also enriched in healthy women from a previous study [71,72, 74]. l-Lysine and methionine are required for the production of l-carnitine which may be important for modulating host immune functions [75]. Other enriched pathways include super pathway of *N*-acetylglucosamine (GlcNAC), *N*-acetylmannosamine (ManNAC) and *N*-acetylneuraminate (NANA) degradation; ethanolamine utilization; and acetylene degradation. These pathways may be involved in microbial competition for nutrient availability. UDP-GlcNAC and UDP-ManNAC are key components of bacterial cell walls. It has been hypothesized that *L. crispatus* encodes glycoside hydrolases which target and destroy other bacterial cell walls [76]. NANA, also known as sialic acid, is present in mucin and utilized by *G. vaginalis* as a carbon source for biofilm formation [77]. Similarly, ethanolamine can also be used as a carbon and nitrogen source by other pathogens and, along with putrescine, is associated with *G. vaginalis*, *F. vaginae* and other anaerobes in women with STIs [78,80]. Finally, acetylene can be degraded into acetate to support growth of many organisms [81]. Acetate has been found to increase bacteriocin production in *Lactobacillus* species [82].

In addition to identifying species enriched in PID cases, shotgun metagenomic analysis allowed us to investigate potential phylogenetic differences in PID cases and controls. Phylogenetic analysis of *L. crispatus*, *L. iners* and *G. vaginalis* did not show strains clustering by case or control status, suggesting that it is the species and abundance that are associated with PID. This contrasts with microbiome studies for preterm births, where different strains of *G. vaginalis* were associated with different risk profiles [83,84].

We also assembled four uncharacterized and uncultured MAGs: GGB3012_SGB4003, GGB34027_SGB48293, GGB4807_SGB6646 (only found in some cases in this study) and GGB1460_SGB2023. These MAGs were all recovered from CST IV individuals, and most were predicted to be strictly anaerobic. They belonged to the *Hungateiclostridiaceae*, *Prochloraceae*, *Lachnospiraceae* and *Bacteroidales*_unclassified families, respectively, and represent new unstudied taxa. One of these unclassified taxa (GB3012_SGB4003) was the fifth most common MAG recovered (slightly more frequently represented in control samples) and, along with GGB1460_SGB2023, has also been detected in other samples [85,86]. To our knowledge, this is the first metagenomic study of cervicovaginal samples from women with PID, and we did not identify any new micro-organisms that could be attributed as responsible for PID.

**Fig. 9.**
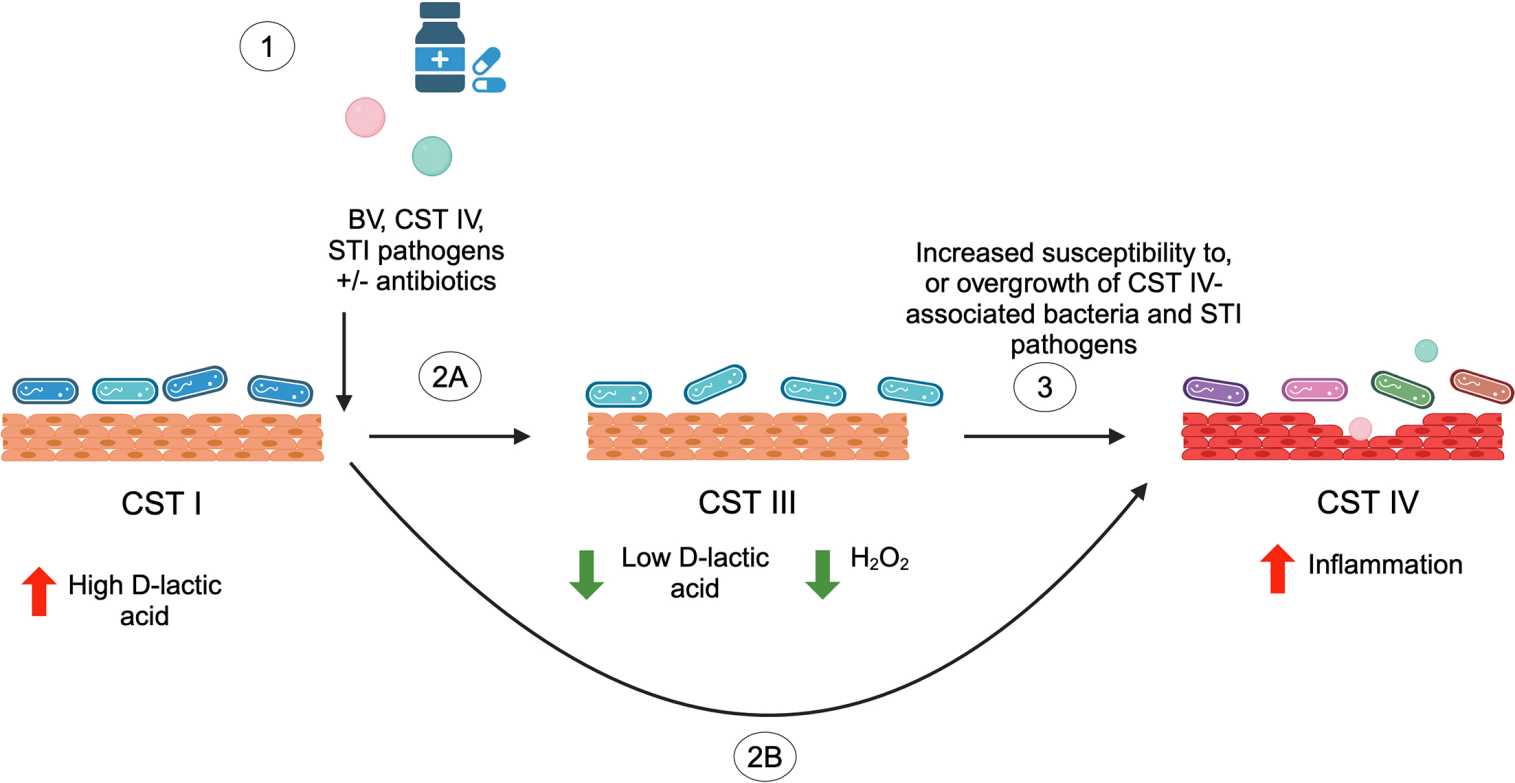
Proposed model of how antibiotics and STI infections or pathogenic organisms lead to dysbiosis and PID. (1) Colonization of STI pathogens such as *C. trachomatis*, or BV CST IV organisms, and/or the use of antibiotics such as DOX to treat STI or other infections, leads to dysbiosis of the cervicovaginal microbiome directly (2B) or indirectly (2A) by first reducing most *Lactobacillus* species except *L. iners*, which are less susceptible to tetracyclines, or by facilitating growth of the more frequently resistant adverse organisms (or both). *L. iners* produces l-lactic acid and lower amounts of H_2_O_2_, increasing susceptibility to colonization by CST IV bacteria and other STIs (3). This results in chronic inflammation, a potentially continued higher burden of dysbiotic and adverse organisms, and may eventually lead to PID.

Overall, our data support a hypothesis that the absolute abundance of the different lactobacilli and corresponding metabolite and active compounds present in combination create an environment that enables or prevents dysbiosis from a microbiota composition perspective. This environment is intrinsically linked to the local tissue immune response, and the interplay impacts the capacity of more harmful micro-organisms to grow to abundance, ascend and vary in proportions along the reproductive tract (as previously reported [87]) and result in PID in the upper reproductive tract (summarized as a hypothetical model in Fig. 9). We observed that PID cases had a modest trend for some factors that could indicate lower Th1 cell response and/or M1 macrophage polarization when measuring gene expression at the cervix. The only potentially upregulated gene (IL-4) is typically associated with Th2 cell response. Th1 cell response is important for protection against intracellular pathogens like *C. trachomatis* by releasing pro-inflammatory cytokine IFN gamma and activating macrophages [88]. Th2 cell responses mediate antibody production through IL-4 [89]. Th2 responses are associated with chronic inflammatory diseases such as asthma and atopic dermatitis [90,92]. However, the immune response data are quite limited in this study, and a much larger sample size would be needed to confirm an upregulation of a Th2 cell response in PID. The data were only significant in the unadjusted t-test and not when corrected for multiple factors. We chose to show these results, as the data could inform the sample size and possible targets for a larger study.

These findings should be interpreted with considerations to the limitations of this study, which include a small sample size, and a lack of longitudinal biospecimen collection that would inform the temporality of microbiome and immune changes with the onset of PID. Further, while strict diagnostic criteria and a chart audit process were followed, the diagnosis of PID is known to be subjective and vary between clinicians [93], meaning there are some risks that not all PID cases (or controls) were accurately diagnosed. Therefore, the biomarkers identified should be considered as preliminary findings that could inform studies with a larger sample size, and perhaps laparoscopic diagnosis.

In conclusion, this study revealed that *L. crispatus* was associated with healthy controls (both in proportional and estimated copy number), whereas *G. vaginalis*, *F. vaginae* and the *Prevotella* genus were associated with PID cases, and a combination of species and their estimated copy numbers and/or proportional abundance was required to differentiate PID from the control. Overall, the study findings support the body of literature that PID is a polymicrobial condition and that low to no abundance of *L. crispatus* could be a contributing factor [14].

## Supplementary material

10.1099/mgen.0.001574 Uncited Supplementary Material 1.

10.1099/mgen.0.001574 Uncited Supplementary Material 2.

## Abbreviations

AML: amoxicillin
AMR: antimicrobial resistance
ANOSIM: analysis of similarity
AUC: area under the curve
AZM: azithromycin
BV: bacterial vaginosis
CSTs: community state types
C_t_: threshold cycle
DOX: doxycycline
LDA: Linear Discriminant Analysis
LEfSe: Linear Discriminant Analysis effect size
MAGs: metagenome-assembled genomes
MRS: Man–Rogosa–Sharpe
NMDS: non-metric multidimensional scaling
PID: pelvic inflammatory disease
qPCR: quantitative real-time PCR
ROC: receiver operating characteristic
STI: sexually transmitted infections
ZOI: zone of inhibition
