# Supplementary material for: Cervicovaginal microbiome composition and absolute quantity are associated with pelvic inflammatory disease
Source: Microb Genom. 2025 Dec 5;11(12):001574. doi: 10.1099/mgen.0.001574 (PMC12680322; doi:10.1099/mgen.0.001574)
Supplement: Uncited Supplementary Material 1. [file mgen-11-01574-s001.pdf]

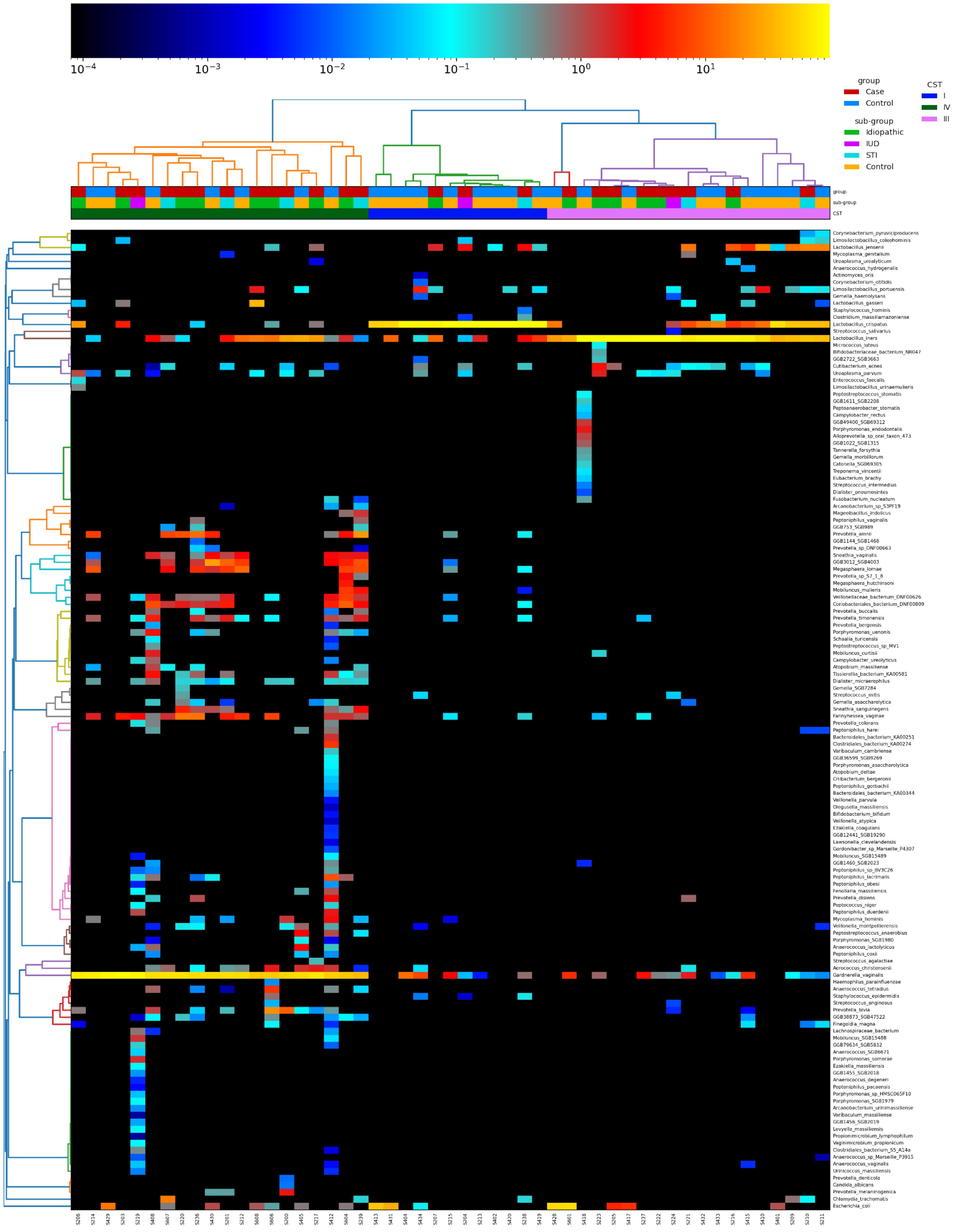

Supplementary Figure 1: Hierarchical clustering dendrogram heatmap for all samples (case, control) and species identified using MetaphlAn4. The sample IDs are on the bottom x-axis, and the species are on the right Y-axis. Group, sub-group metadata and assigned CST are shown on the top, with the legend on the bottom.

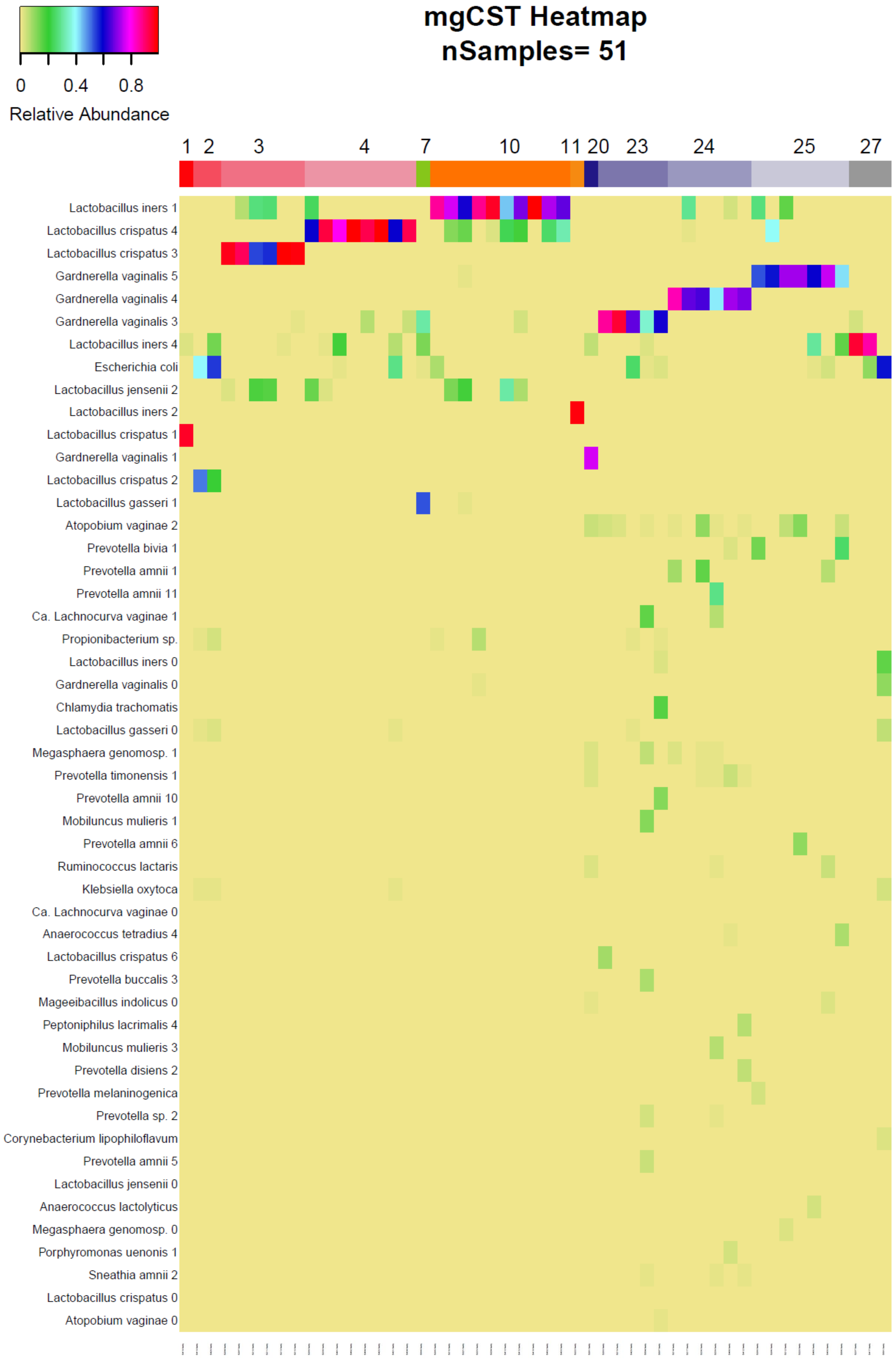

Supplementary Figure 2. Hierarchical clustering dendrogram heatmap for all samples and species identified using mgCST-classifier. The sample IDs are on the bottom x-axis, and the species are on the right Y-axis. Assigned mgCSTs are shown on the top.

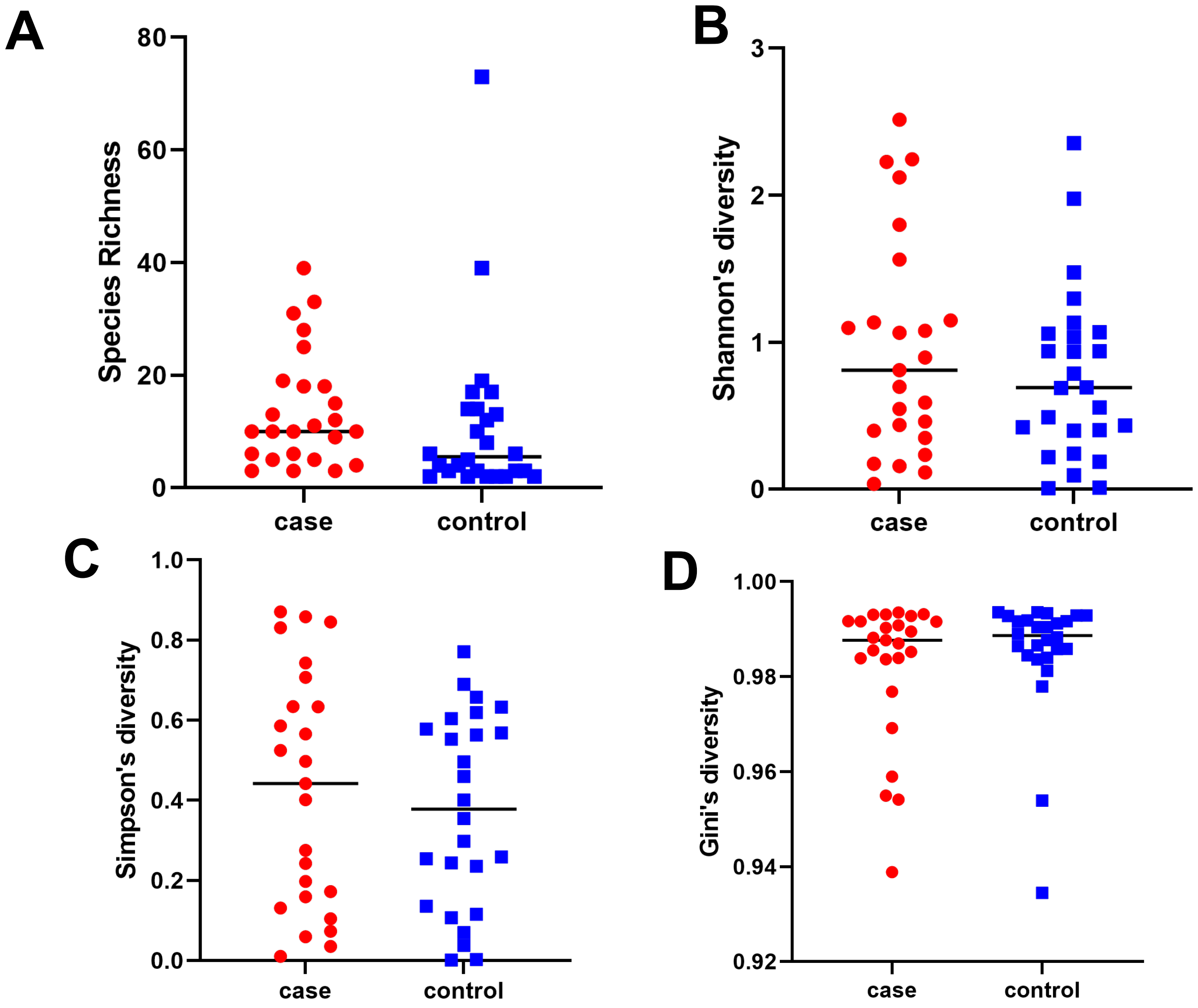

Supplementary Figure 3: Alpha diversity measures compared between cases and controls.

The red and blue dots represent case and control samples, respectively. A represents species richness, B represents Shannon's diversity, C represents Simpson's diversity, and D represents Gini's diversity. The horizontal line represents the mean.

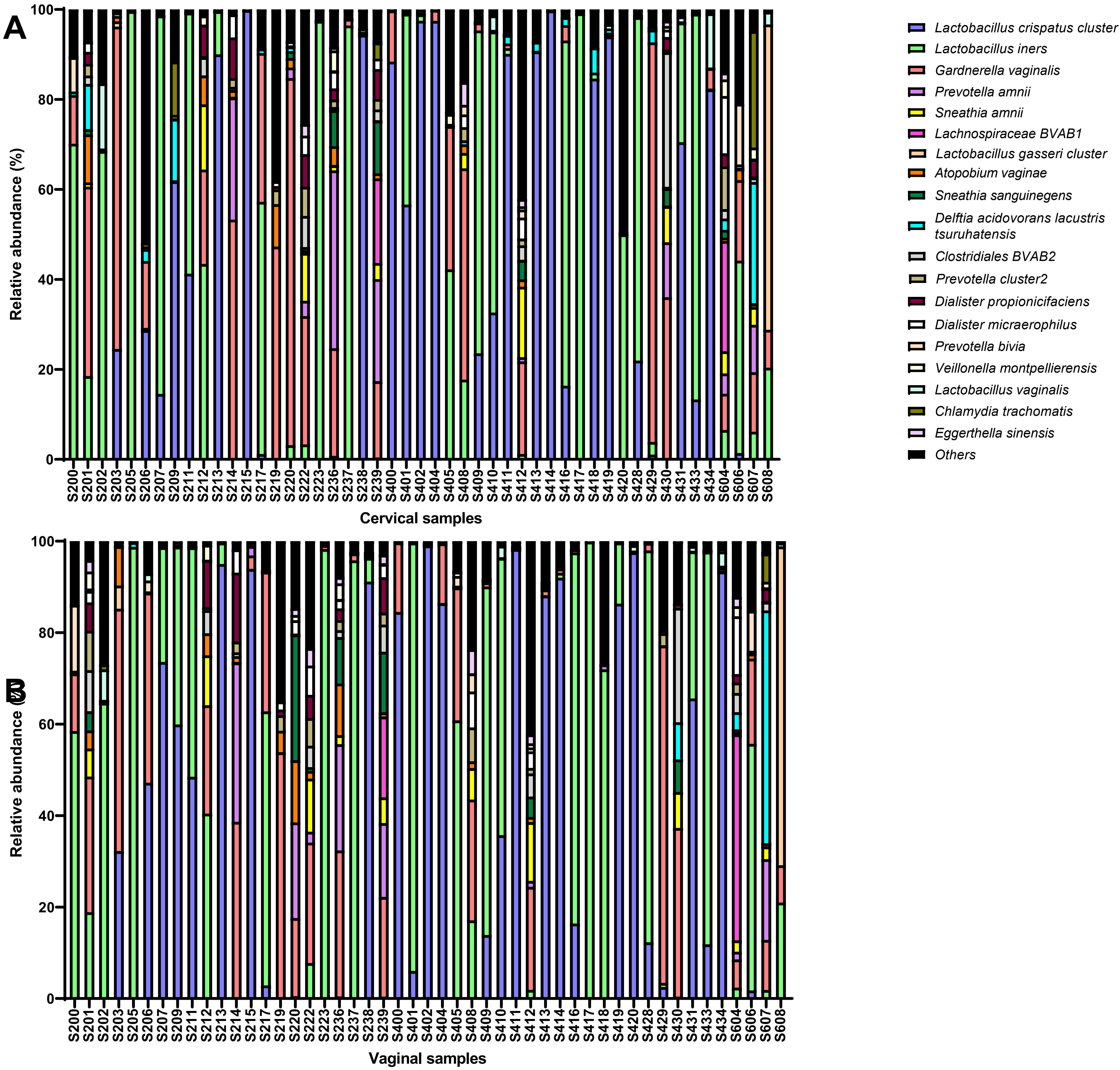

Supplementary Figure 4: Stacked bar graphs showing relative abundance for the top 30 species in (A) cervical and (B) vaginal samples. The x-axis displays each individual participant with the left y-axis showing the two sampling sites. The left y-axis displays the relative abundance score from 0 to 1.

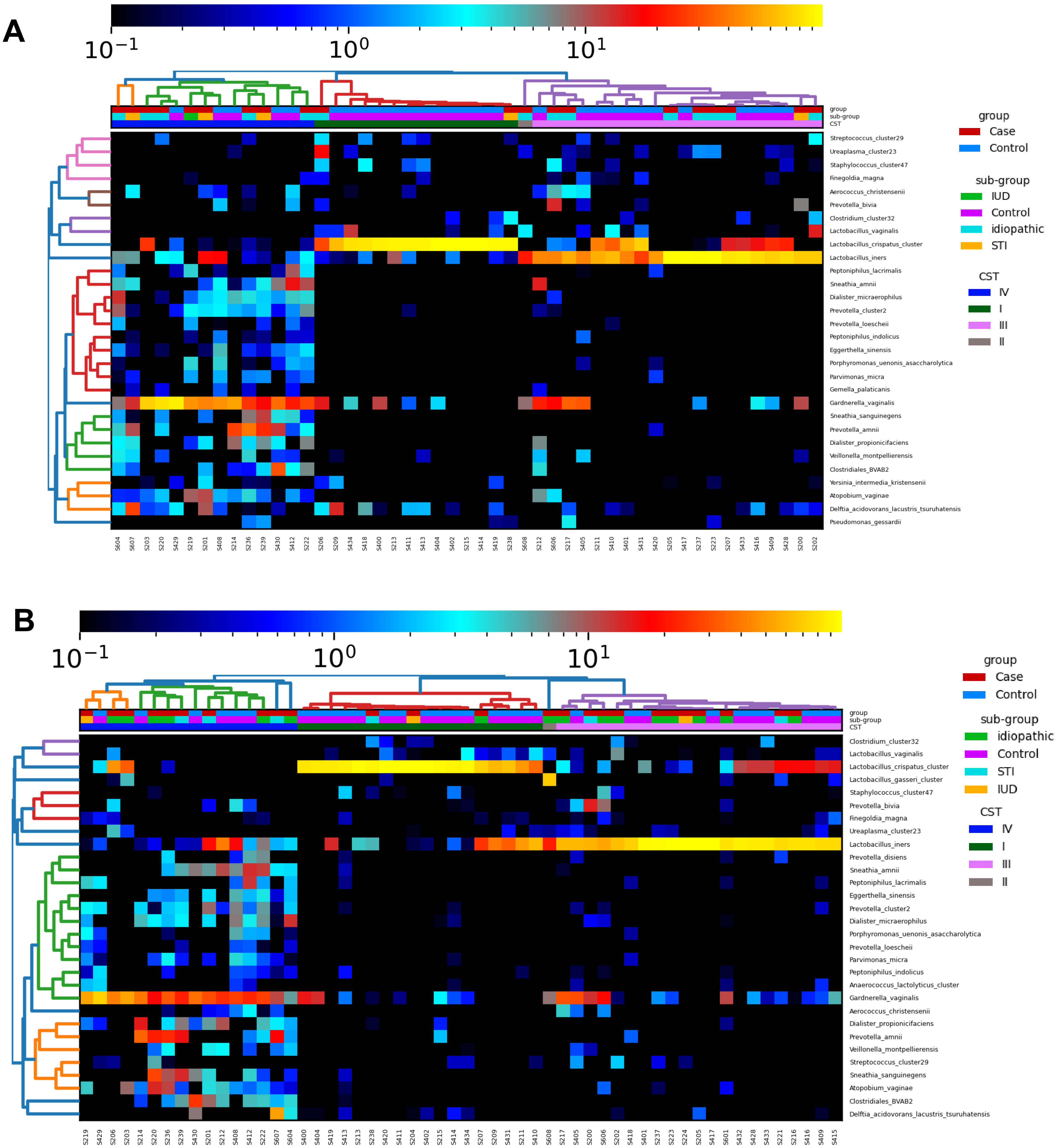

Supplementary Figure 5: Cluster linkage dendrogram heat map of top 30 most abundant species in participant (A) cervical and (B) vaginal samples analysed by 16s rRNA sequencing. The relative abundance scale (on top right) shows reads from 0 to 2000 reads (samples were rarefied to 2000 reads), and colour scale is white to red (red is highest relative abundance and white lowest). The heat map is arranged by a column cluster linkage dendrogram followed by annotation with corresponding legends on the top and right respectively.

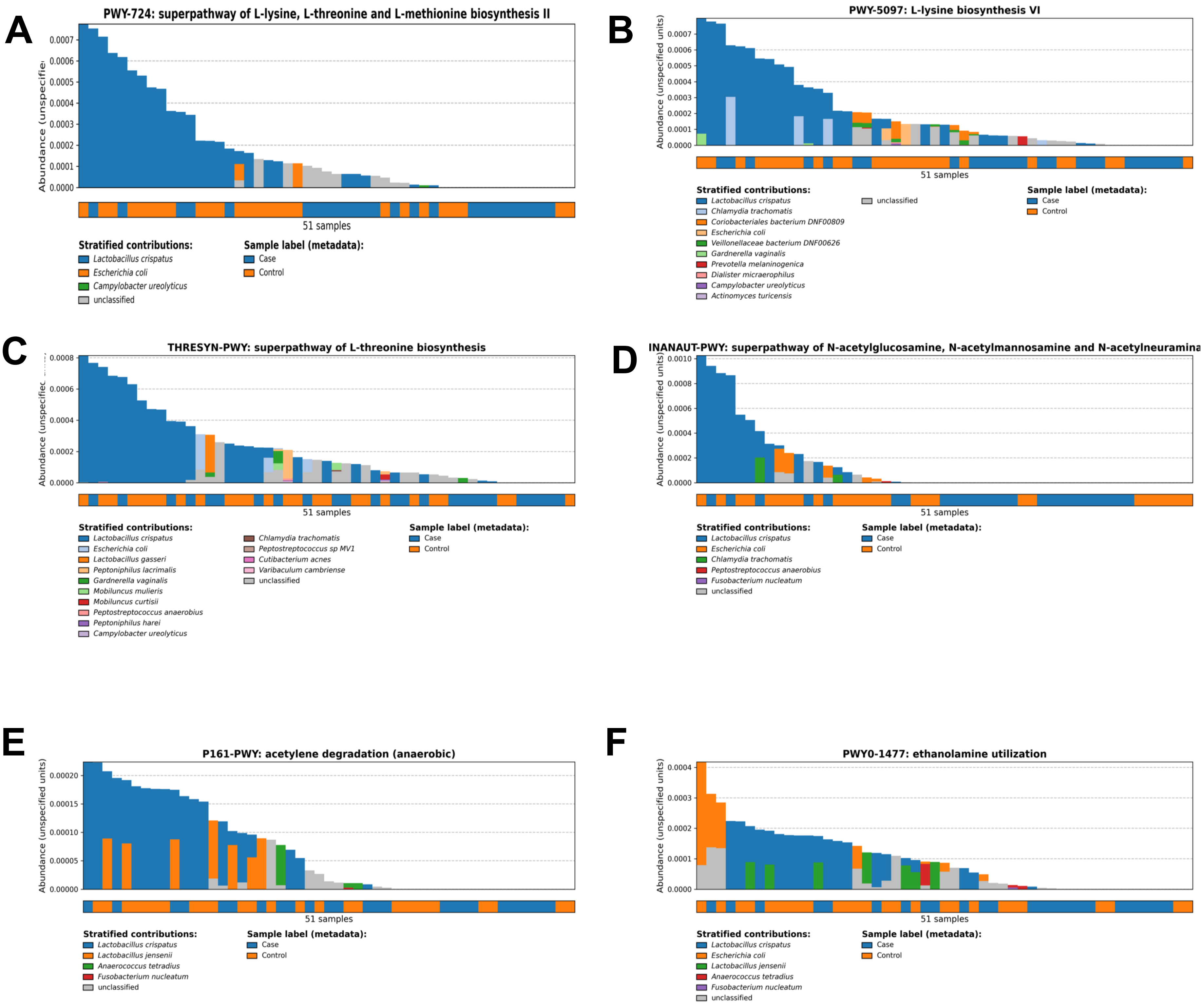

Supplementary Figure 6: Bar plots of significant pathways associated with controls and their contributing species. For each panel, the y-axis represents the abundance of contributing species in each pathway, and the x-axis represents each sample. The colour of the stratified bar plot represents individual contributing species with the species identification in the legend below. The bottom bar represents the group metadata (cases in blue, control in orange). (A) super pathway of L-lysine, L-threonine, and L-methionine biosynthesis, (B) L-lysine biosynthesis VI, (C) superpathway of L-threonine biosynthesis, (D) super pathway of N-acetylglucosamine, N-acetylmannosamine and N-acetylneuraminate degradation, (E) acetylene degradation (anaerobic), and (F) ethanolamine utilization.

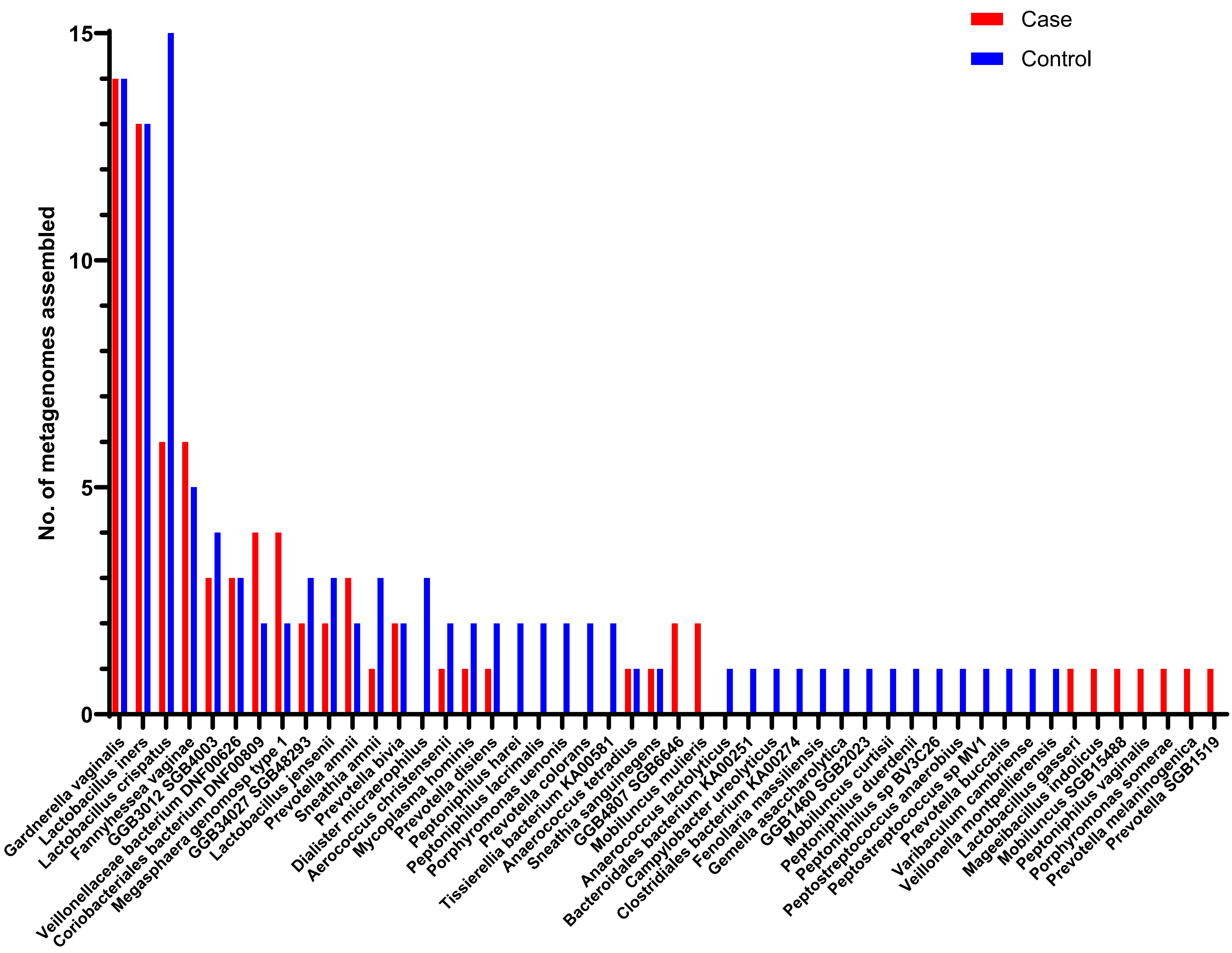

Supplementary Figure 7: Number of MAGs assembled from PID and control samples for each species.

The Y-axis represents the number of MAGs assembled, and the X-axis represents the labels of individual species. MAGs assembled from the cases and controls are represented by red and blue colours, respectively.

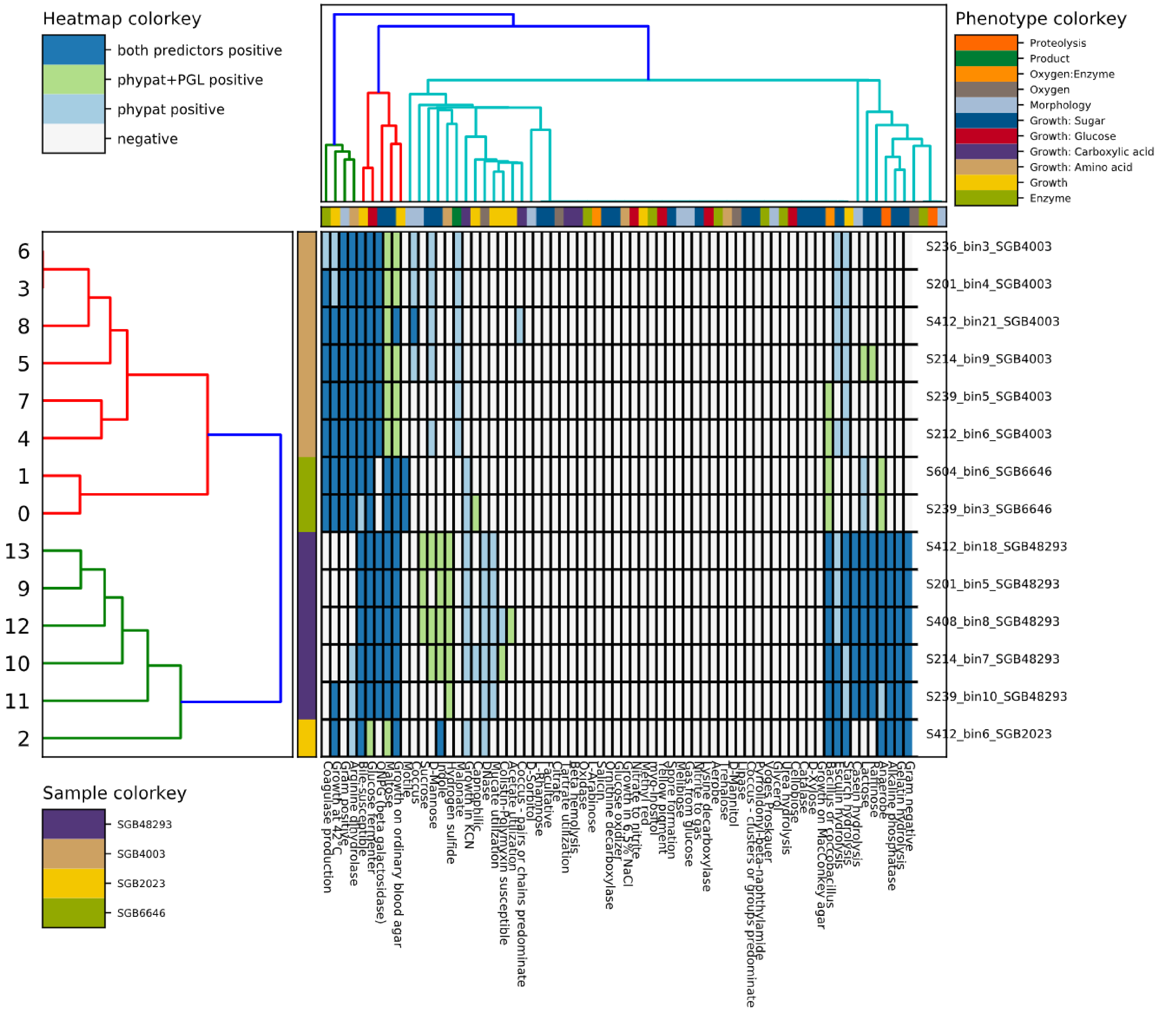

Supplementary Figure 8: Traitar predicted microbial characteristics of uncharacterised organisms identified in the study. The right Y axis indicates the predicted organism and source sample. The x axis indicates predicted phenotypic traits, and the colour code key for these is shown at the top right and left. Cluster linkage dendogram analysis of the metagenome (left) and predicted phenotypes (top).

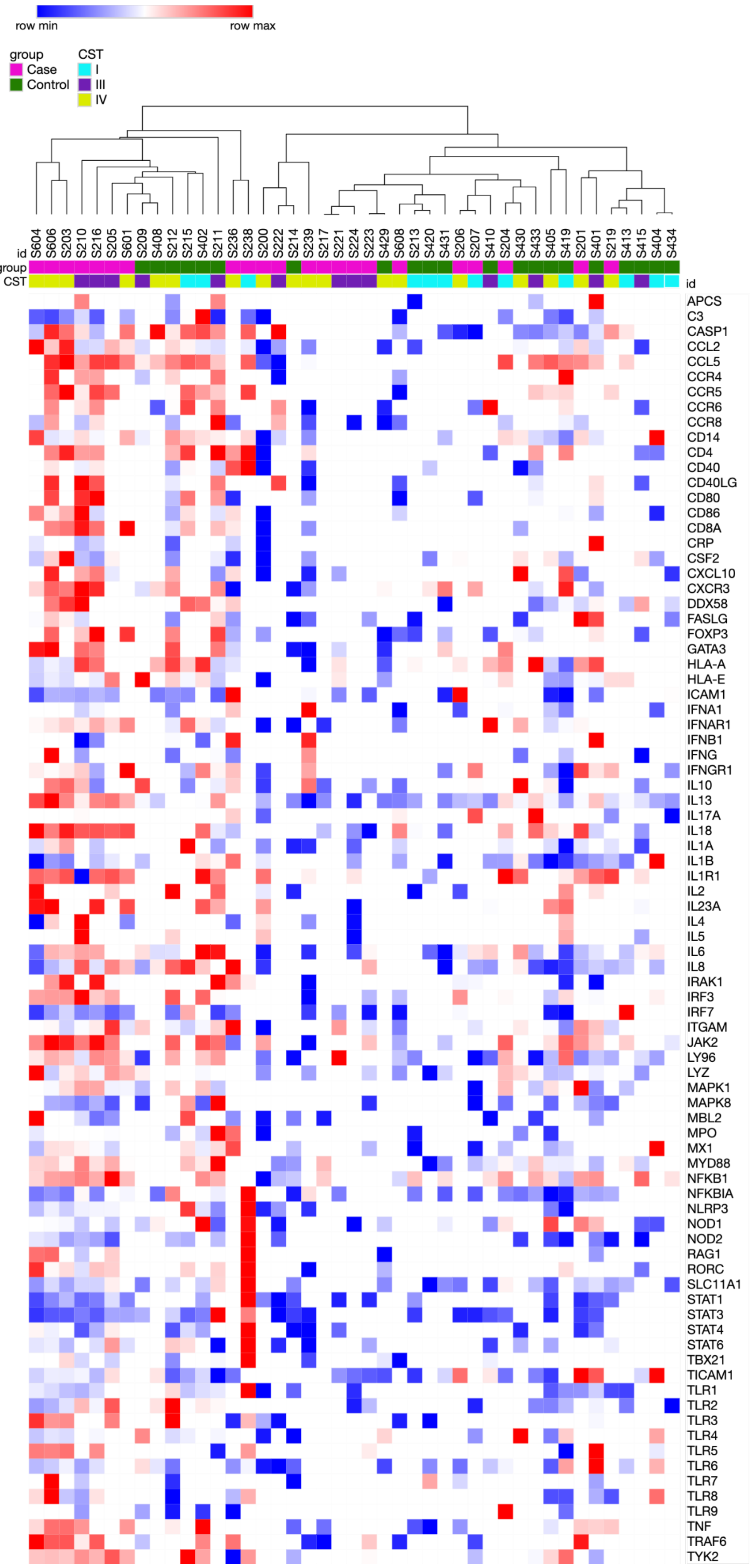

Supplementary Figure 9: Cluster dendrogram heatmap of the relative gene expression of human innate and adaptive immune response associated genes from case and controls. The heatmap was created using 2^-deltaCt values. The scale on the top left side represents the gene expression level. Red indicates upregulation, white indicates below the limit of detection or no expression, and blue indicates downregulation of genes. The group and CST are shown in the metadata panel. On the x-axis are the sample IDs, and the y-axis are the immune-associated genes.

A

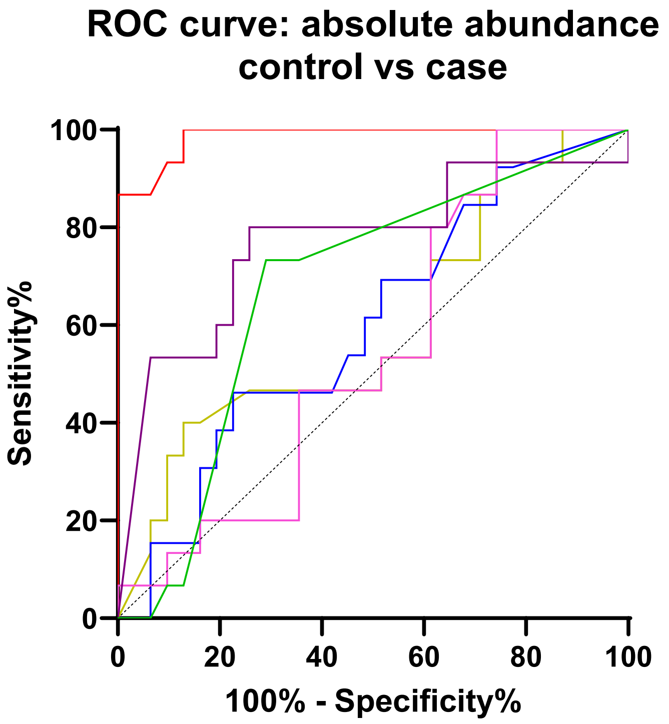

Species	AUC	p-adj value	95% CI	Opt. threshold	SE	SP
L. iners	0.60	0.372	0.42-0.78	530203.9	0.46	0.63
L. crispatus	0.99	0.006	0.96-1	3255394	1.0	0.87
G. vaginalis	0.60	0.372	0.42-0.78	96693.8	0.4	0.77
F. vaginae	0.67	0.120	0.51-0.84	8965.8	0.73	0.71
M. genitalium	0.55	0.61	0.38-0.72	35401058	0.8	0.35
Prevotella spp	0.77	0.009	0.61-0.93	294504.3	0.8	0.74

B

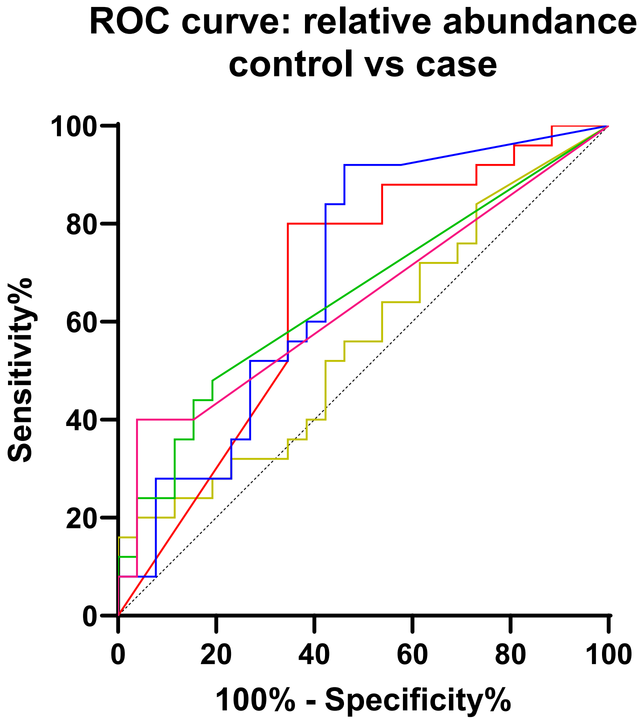

Species	AUC	p-adj value	95% CI	Opt. threshold	SE	SP
L.iners	0.56	0.451	0.4-0.72	93.02	0.2	0.96
L. crispatus	0.67	0.085	0.52-0.82	8.78	0.8	0.65
G. vaginalis	0.69	0.085	0.54-0.84	0.022	0.92	0.53
F. vaginae	0.65	0.104	0.49-0.8	0.017	0.48	0.8
U. parvum	0.64	0.104	0.48-0.79	0.03	0.4	0.96

C

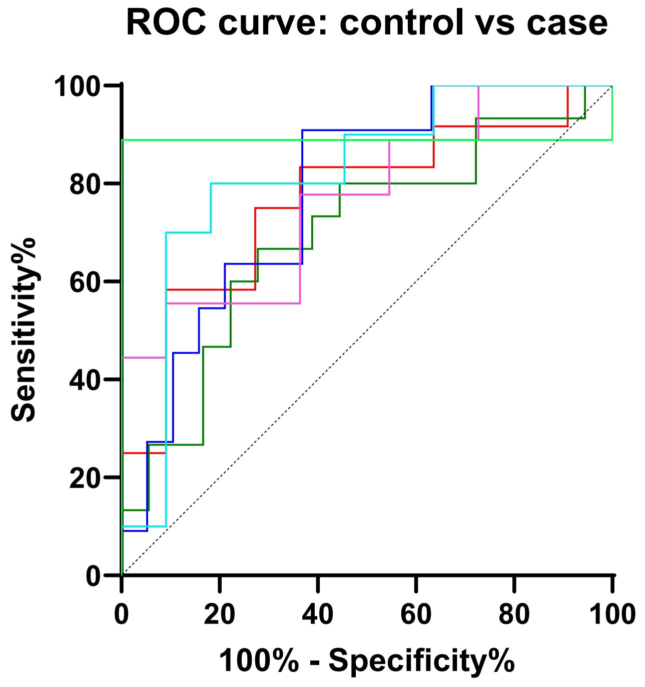

Gene	AUC	p-adj value	95% CI	Opt. threshold	SE	SP
IFNGR1	0.77	0.06	0.54-0.96	0.05	0.58	0.9
LY96	0.69	0.07	0.51-0.88	0.22	0.68	0.68
SLC11A1	0.78	0.03	0.61-0.94	0.14	0.9	0.63
STAT4	0.77	0.06	0.55-0.98	0.01	0.55	0.9
TNF	0.81	0.03	0.62-1.00	0.03	0.8	0.81
IL4	0.89	0.22	0.68-1.00	0.01	0.88	1.00

Supplementary Figure 10. ROC curve to identify potentially predictive microbial markers of PID. ROC curve to evaluate sensitivity and specificity of enriched species (A) using absolute abundance data from qPCR to detect PID, (B) using shotgun metagenomic data to detect PID, and (C) using immune gene expression data to detect PID. The y-axis represents sensitivity, and the x-axis represents false positives (100- specificity). Each colour line represents the ROC curve of an individual tax. Area under the curve (AUC), p-adjusted value using Benjamini-Hochberg method, 95% confidence interval (CI), optimal threshold, sensitivity (SE) and specificity (SP) for each enriched taxa is shown on the right side of the graph.

A **Combiroc: case vs control**

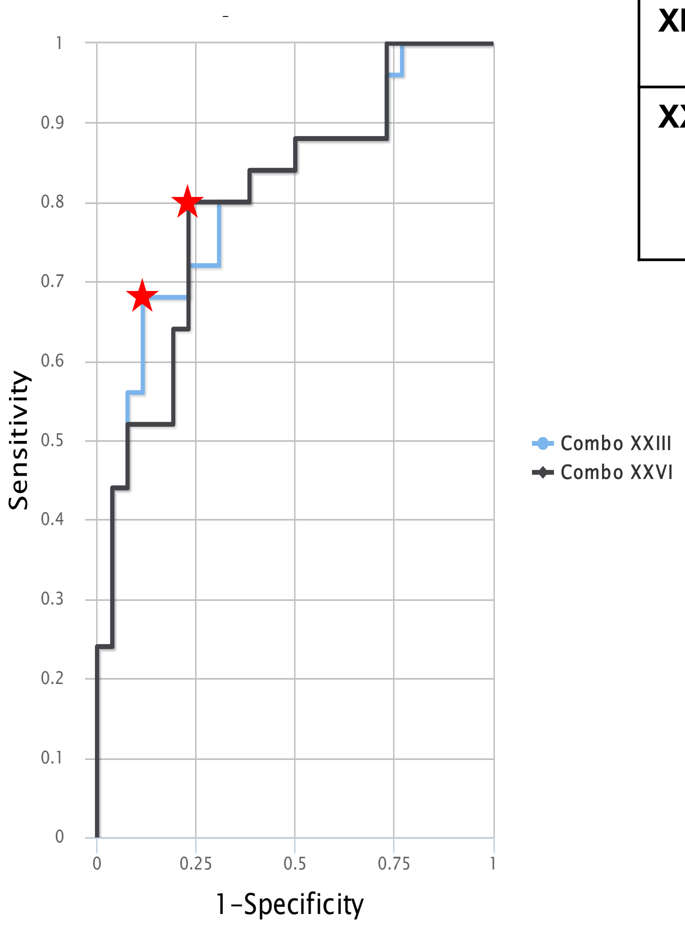

Combo	Species	AUC	Opt. threshold	SE	SP
**XIII**	***L. iners, L. crispatus, F. vaginae, U. parvum***	**0.81**	**0.56**	**0.68**	**0.85**
**XXVI**	***L. iners, L. crispatus, G. vaginalis, F. vaginae, U. parvum***	**0.8**	**0.44**	**0.8**	**0.76**

Supplementary Figure 11: CombiRoc curve for combined microbiome panels testing for differences between control vs case. The red star on the curve represents the optimal threshold value for each ROC curve.
